# User experience design methodologies for developing a tele-round platform in public intensive care units in northern and northeastern Brazil

**DOI:** 10.3389/fdgth.2026.1713349

**Published:** 2026-04-08

**Authors:** Ana Beatriz Frade Moura, Fabiane Raquel Motter, Lyvia Mota da Silva, Izadora Coelho da Silva, William Aparecido Santos Silva, Beatriz de Faria Leao, Shoraya Virginio Carneiro Dal Col, Sabrina Dalbosco Gadenz

**Affiliations:** 1 Diretoria de Compromisso Social, Hospital Sírio-Libanês, São Paulo, SP, Brazil; 2 Instituto de Ensino e Pesquisa, Hospital Sírio-Libanês, São Paulo, SP, Brazil

**Keywords:** intensive care units, software design, telemedicine, user-centered design, user-computer interface

## Abstract

**Introduction:**

Designing digital health solutions for critical environments like intensive care units (ICUs) is challenging, especially in resource-constrained settings. The integration of user experience (UX) design methods into digital health development may improve alignment with clinical workflows, reduce barriers to adoption, and enhance perceived usefulness.

**Objective:**

To apply user experience design methodologies to develop the interface of a telemedicine platform intended to support multidisciplinary tele-rounds in public ICUs in Northern and Northeastern Brazil.

**Methods:**

We conducted a methodological study from February to May 2022, using the four-stage Double Diamond design model: Discover, Define, Develop, and Deliver. The design process was embedded within a Tele-ICU program implemented through the Brazilian Unified Health System (SUS), supporting public ICUs across the North and Northeast regions of the country. Design activities included desk research, rapid ethnography, benchmarking, development of personas and empathy maps, situational diagnosis of participating ICUs, user journey mapping, wireframing, and heuristic-based usability evaluation.

**Results:**

The primary outcome was the successful development and implementation of the “Mangará Digital” tele-round platform. The user-centered process directly informed key features designed to address identified user “pains”, such as time pressure and lack of process standardization. These features included at-a-glance patient summary cards, a visual ICU bed map, and integrated checklists.

**Conclusion:**

This study demonstrates that UX design methodologies can effectively guide the development of telemedicine platforms tailored to the realities of public ICUs in underserved regions. Explicit consideration of geographic, organizational, and infrastructural constraints is essential to ensure usability, adoption, and sustainability of digital health solutions in resource-constrained intensive care settings.

## 1 Introduction

Telehealth interventions in intensive care units (ICUs) aim to optimize care for critically ill patients while strengthening training and decision support for remote ICU teams. Evidence suggests that tele-ICU models can reduce mortality and length of stay, particularly in services facing difficulties recruiting and retaining specialized personnel (1–5). However, translating telehealth promise into routine practice depends not only on connectivity and clinical protocols, but also on the usability and reliability of the digital tools that support daily work. Despite rapid advances in Information and Communication Technologies (ICTs) in healthcare, developing electronic medical record (EMR) systems with intuitive, user-friendly interfaces that perform reliably in the high-acuity ICU context remains challenging (1, 3, 5, 6).

Integrating user experience (UX) design methods into telehealth development can improve alignment with clinical workflows, reduce adoption barriers, and increase perceived usefulness. Although UX methodologies have been applied to telehealth solutions across multiple clinical domains, most published work still comes from high-income countries (7–15). In Latin America, the application of structured UX frameworks to telehealth remains limited. In particular, there is little literature describing the use of established design frameworks, such as the Double Diamond model, to develop and refine software for tele-round documentation in ICU telehealth programs (13, 16, 17).

This evidence gap is particularly important in low- and middle-income countries (LMICs), where telehealth implementation often occurs in settings characterized by limited digital infrastructure, constrained implementation capacity, and workforce shortages in critical care (18). In Brazil, these challenges are particularly pronounced in the North and Northeast regions, where access to board-certified intensivists is limited (19). To address these inequities, national strategies have been implemented, including the “Qualification of Care in Intensive Care through Telemedicine” project (Tele-ICU project) (20).

This initiative is conducted through the Institutional Development Support Program of the Unified Health System (PROADI-SUS), a collaborative program between the Brazilian Ministry of Health and leading philanthropic hospitals. Within the Tele-ICU project, Hospital Sírio-Libanês (HSL) provides clinical expertise and technological infrastructure to support public ICUs in the North and Northeast regions selected by the Ministry of Health based on regional inequities and specialist shortages in specialized intensive care, using a hub-and-spoke model (20). However, for remote support to translate into consistent and scalable clinical practice, fit-for-purpose digital tools are also required to structure tele-round workflows, organize clinical information, standardize documentation, and reduce cognitive load for teams working under sustained time pressure.

In this context, this study applies user experience (UX) design methodologies to develop the interface of a telemedicine platform intended to support multidisciplinary tele-rounds in public ICUs in Northern and Northeastern Brazil.

## 2 Methods

This section describes the study design, implementation setting, multidisciplinary design team, and the stepwise design activities organized according to the Double Diamond framework.

### 2.1 Study design

We conducted a methodological study using a human-centered design approach to design an interface for the tele-round recording system (Mangará Digital) from February 2022 to May 2022. This project was approved by the research ethics board at the Hospital Sírio-Libanês (Number: 6.215.082).

### 2.2 Setting

The Tele-ICU project (NUP 25000.177384/2021-11) was established to support medical and multidisciplinary teams in participating intensive care units (ICUs) through telemedicine.

As part of the project, Hospital Sírio-Libanês (HSL) supported 15 public ICUs located in the states of Pará, Piauí, Maranhão, and Acre. Participating hospitals were selected by the Ministry of Health, prioritizing ICUs in Brazil's North and Northeast regions where access to specialized intensive care is limited. HSL acted as the centralized hub, providing remote support to participating ICUs through tele-rounds and complementary implementation activities. The comprehensive implementation and multi-modal intervention of the Tele-ICU project, including the detailed operational model of daily multidisciplinary tele-rounds, educational initiatives, and protocol development, are described in a previous study (20). Furthermore, all participating units adhere to the same national clinical and infrastructure regulations, allowing for the scalability and comparability of the digital health interventions across different states within the Brazilian territory.

### 2.3 Design team

The design team consisted of a UX designer, an assistant with knowledge of customer service and communication processes, a nurse with expertise in intensive care, a project manager with experience in digital health, and a developer. The involvement of each member is outlined in Supplementary Material S1.

### 2.4 Design process

**Figure 1:**
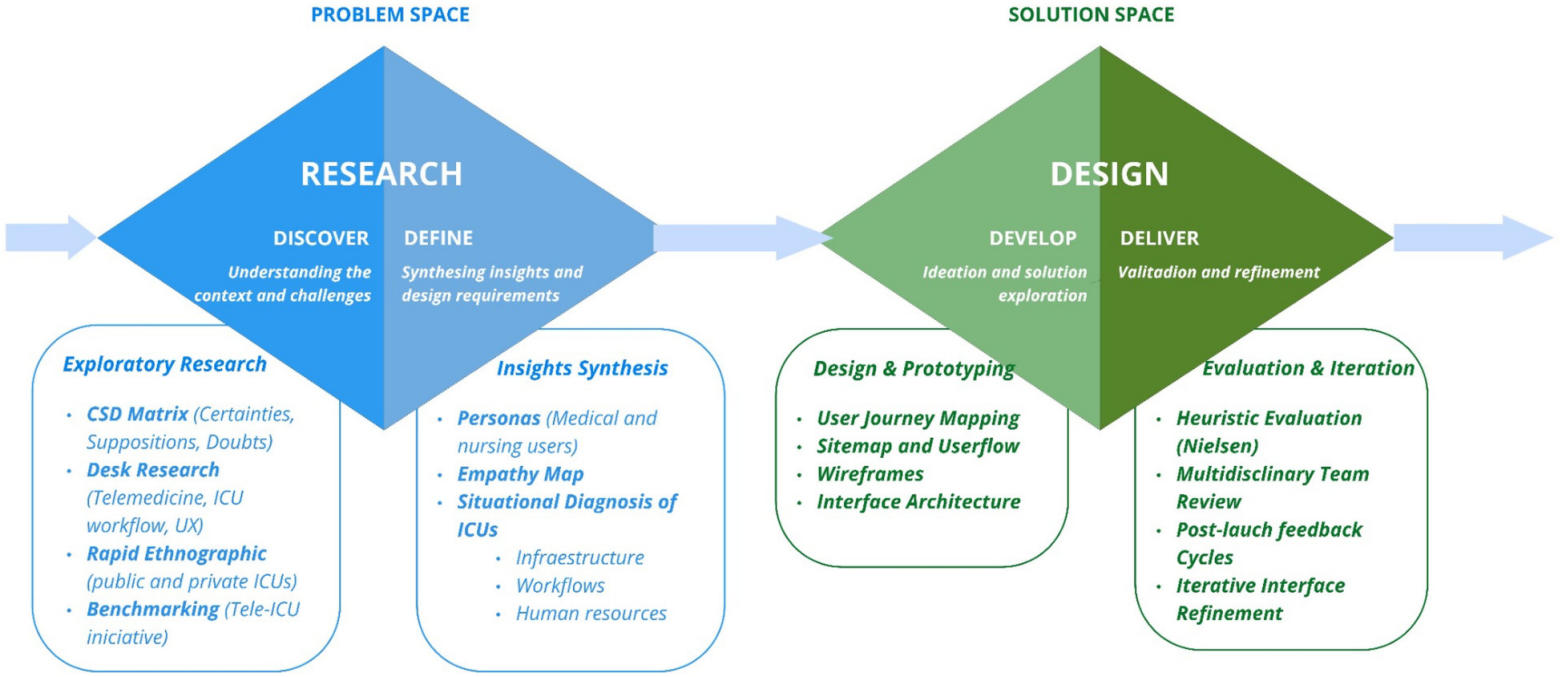
Application of the double diamond framework in the development of the mangará digital platform.

The Double Diamond methodology guided the design process through four stages: Discover, Define, Develop, and Deliver (21). The Discover and Define stages focus on understanding the problem through contextual exploration, data gathering, and synthesis of insights into a clear problem statement. The Develop and Deliver stages involve ideation, prototyping, and testing to generate, refine, and implement practical and effective solutions. Figure 1 provides an overview of the double diamond steps; further details regarding the methodological process are provided in Supplementary Material S1.

#### 2.4.1 Discover

The Discover phase aimed to gather diverse information to immerse the design team into the problem context (21). In this study, the methods employed during this phase included the CSD Matrix (Certainties, Suppositions, and Doubts), Desk Research (DR), Rapid Ethnography, and Benchmarking. This stage aimed to characterize the clinical context, identify early assumptions and uncertainties, and collect evidence regarding workflows and existing tele-ICU practices.

##### 2.4.1.1 Step 1-CSD matrix (certainties, suppositions and doubts)

To initiate the discovery process and align the team's initial understanding of the project scope, we applied the CSD matrix method. This framework synthesizes a project's knowledge base by categorizing information into three domains: Certainties, Suppositions, and Doubts (22). This tool serves as a dynamic decision-support instrument that enables multidisciplinary teams to prioritize research efforts and mitigate risks by addressing ambiguities transparently during the early stages of development (22, 23).

A meeting was conducted in February 2022 between design team members to construct the CSD Matrix. The theme of the matrix was “tele-round.” The session leveraged the Miro interactive platform for real-time data collection and thematic organization. Insights were systematically categorized according to the following criteria:

1. Certainties: Information derived from the formally approved institutional work plan and the research team's cumulative expertise in digital health deployment.
2. Suppositions: Hypotheses formulated on the basis of the existing literature and knowledge in the intensive care (IC) field, particularly regarding the feasibility and clinical applicability of the tele-round model within the existing workflow.
3. Doubts: Questions related to local contextual specificities, diverse user profiles, and unexplored technological possibilities that required further empirical investigation.

##### 2.4.1.2 Step 2-desk research (DR)

Following the initial alignment, we performed comprehensive desk research to ground our assumptions in existing literature and identify evidence-based practices. Desk research is a question-driven review of existing materials—often including non-scientific sources such as intervention descriptions, management reports, and stakeholder artifacts—to build a broad understanding of the topic (24).

In this study, desk research was conducted to situate the project within the current evidence base and prevailing technological practices in telemedicine and intensive care. This step informed decision-making by identifying design strategies, engagement mechanisms, and system requirements described in the literature and in real-world implementations.

To operationalize this step, initially, a meeting was conducted between design team members to define the research topics based on assumptions and doubts raised in the previous step. A literature search was conducted between the 15th and 25th of March in research databases (Research Gate, SciELO Brazil, Critical Care Nursing Quarterly, JMIR Publications) and websites (Google) using the following keywords: engagement, ICU, telemedicine, tele-round, user feedback, integration, health system, data sharing and communication. Searches were limited to English and Portuguese languages.

Methodological studies, case studies, and interventions related to digital products that addressed the following topics were included: user engagement, technological structure, communication strategies, multidisciplinary teams, data collection, and sharing. The inclusion of articles was not restricted to the digital health area; thus, studies from other areas that were compatible with the research topics and those that described new technologies were included.

We excluded studies that applied design approaches, but the brief descriptions of their design work needed to be more comprehensive to meet the proposed objective. The collected data were registered in Excel. Finally, a discussion group was performed during a meeting to discuss the findings and seek insights to support the project.

##### 2.4.1.3 Step 3-rapid ethnography (REA)

To gain a deeper understanding of the ICUs' physical environment and social dynamics, we used rapid ethnography to observe real-world workflows. Rapid ethnography combines focused fieldwork and rapid synthesis to generate contextual insights within constrained timelines (25). In this study, we conducted rapid ethnographic research in the ICUs of two hospitals—one public and one private—to explore the behavior patterns of multidisciplinary teams, ICU routines, and the similarities and differences between the two settings. The ICUs were selected by convenience sampling. The UX designer conducted two in-person visits, each lasting five hours.

At each site, participant observation and semistructured interviews with staff members were used to examine the local context (number of beds, type of patients, preparation for the rounds, registration of patient data), human resources (composition of the team, routine of each professional, work schedule), and ICU processes (digitalization of data, collection of indicators, visualization of exams, recording of rounds, use of a standardized checklist, existence of Standard Operating Procedures, and multidisciplinary team). The authors developed a semistructured interview guide for conducting the interviews (Supplementary material S2). The data collected were manually recorded and digitized in Notion.

##### 2.4.1.4 Step 4-benchmarking

To complement the field observations and learn from similar initiatives, we conducted benchmarking interviews to identify best practices and potential implementation pitfalls. Benchmarking is a structured comparative approach used to examine existing initiatives in order to identify effective practices, operational strategies, and design solutions applicable to a given context (26). In this study, benchmarking was conducted through semi-structured video interviews with two project managers who had led the implementation of telemedicine initiatives in Brazilian public ICUs. The interview included the following topics: structure of the project (platforms used, situational diagnosis, and video communication technologies), processes (agenda management, standardization of care, routines with the ICUs), and human resources (users' adherence, interaction with technology and definition of roles). A semi-structured interview guide developed by the authors was used to ensure consistency across interviews and is provided in Supplementary Material S3.

#### 2.4.2 Define

The Define phase focused on synthesizing insights gathered during discovery into actionable design directions. By translating observed challenges into clear priorities, we established the user archetypes and workflow requirements needed to guide interface decisions. This phase was comprised of two key steps: personas and empathy maps and situational diagnosis.

##### 2.4.2.1 Step 5-personas and empathy maps

Based on the data collected during the Discover phase, we developed user archetypes to humanize the design process and maintain a focus on user needs. To support this step, two empathy maps were created, one for medical users and another for nursing users. Each map was organized into six domains: (1) what the user thinks and feels; (2) what the user sees; (3) what the user hears; (4) what the user says and does; (5) pains; and (6) needs. The content of each domain was informed by the evidence and insights generated in the previous stages of the methodology. The empathy maps were developed collaboratively by the UX designer and a nurse during a 2-hour video meeting using the Miro platform.

##### 2.4.2.2 Step 6-situational diagnosis

To assess the technical readiness and infrastructural constraints of each implementation site, a situational diagnosis was conducted. This step aimed to characterize the infrastructure, workflows, and staffing of participating ICUs in order to align the interface design with feasibility conditions.

We conducted a situational diagnosis for each participating ICU in the project. A questionnaire (Google Forms) developed by team members (Supplementary Material S4) was sent to each ICU coordinator. It was organized into five sections: 1. general information about the hospital (hospital's size, availability of exams and medical devices on-site); 2. ICU infrastructure (availability of x-ray and ultrasound machines at the bedside, level of care provided to COVID-19 patients, and usage of electronic and/or physical medical records); 3. human and material resources (size, workload, and diversity of the ICU care team); 4. workflows (availability of protocols and daily census); 5. ICU indicators. A descriptive analysis was performed for each of the variables collected. The collected data were analyzed across three categories—strengths, areas for improvement, and bottlenecks—to identify discrepancies between the maturity of clinical processes and gaps in technological infrastructure, thereby guiding the prioritization of interface requirements.

#### 2.4.3 Develop (ideation)

During the Develop phase, insights gathered from previous stages were transformed into concrete design solutions. Collaborative ideation activities were used to map user interactions, define the information architecture, and prototype interfaces aligned with ICU workflows. This phase comprised three steps: user journey mapping, sitemap and user flow design, and wireframe construction.

##### 2.4.3.1 Step 7-user journey mapping

User journey mapping was used to visualize the end-to-end clinical workflow and identify key touchpoints between users and the system. This step mapped tasks across the pre-round, round, and post-round phases, supporting the identification of interface priorities and design requirements.

The journey map was developed in two stages. First, an initial version was produced through collaborative brainstorming during two meetings. Second, this version was refined into a more detailed representation of the workflow. The resulting outputs were reviewed and discussed during six virtual meetings involving team members, a project coordinator, and a data specialist.

##### 2.4.3.2 Step 8-sitemap and userflow

By translating the user journey into a logical digital structure, a sitemap and user flow were developed to define the platform's navigation and information hierarchy.

Using Figma, the UX designer translated insights from the ideation stage into the platform's core modules, including the home screen with patient “cards” displaying summarized information, the medical record, the indicator entry page, and the patient and hospital registration lists. These components were organized according to usability principles and informed by Nielsen's heuristic framework.

##### 2.4.4.3 Step 9-wireframes construction

Building upon the information architecture, the wireframing process focused on designing the visual layout and interface interactions to ensure usability. This step involved the development of initial prototypes and the proposal of the interface structure. The process was conducted in Figma under the guidance of the UX/UI designer. Additionally, a project specialist with expertise in data and digital health and a specialist in health intelligence and technology provided valuable insights during this stage. The wireframe construction phase lasted approximately four weeks and consisted of four synchronous meetings to discuss functionalities and interface requirements and asynchronous activities for production.

Concurrently, the platform development stage commenced with a focus on defining the databases. Two programmers were assigned to develop the screens, and the Minimum Viable Product (MVP) was delivered within three months.

#### 2.4.4 Deliver

The final convergent phase focused on validating and refining the proposed solution before its official launch. This stage emphasized assessing the platform's usability and technical feasibility under real-world implementation constraints through Step 10, testing and validation.

##### 2.4.4.1 Step 10-testing and validation

This step involved a validation process to evaluate the usability and technical viability of the proposed solution prior to full-scale deployment. Ideally, this phase would include formal usability testing with end-users. However, due to an accelerated project timeline and logistical constraints, an adapted approach was employed. We used Nielsen's heuristics to evaluate the usability of wireframes (6). In addition, meetings were held with the Tele-ICU project team (doctors, nurses, and physiotherapists) to discuss the visualization of the screens and technical and functional details of the solution. The suggestions were registered on the Figma platform. To complement this evaluation, a process was established for the systematic collection of post-launch feedback. This involved monthly meetings to evaluate the platform's features and usability, serving as a validation mechanism in a real-world use scenario.

## 3 Results

The user-centered design process enabled the development and implementation of the Mangará Digital platform, which was launched on June 21, 2022. By November 30, 2023, the platform had supported 3,971 tele-rounds involving 5,471 distinct patients. The insights generated across each stage of the Double Diamond framework, and their implications for the platform's design, are presented below.

### 3.1 Discover

During the discovery phase, consistent patterns were identified across ethnographic observations, the desk research and benchmarking analysis. ICU workflows were characterized by severe time constraints, fragmented clinical information dispersed across paper records, verbal communication, and messaging applications, as well as limited process standardization. Collectively, these factors contributed to cognitive overload, uncertainty during rounds, and dependence on informal workarounds. In response, three high-priority design requirements were defined: rapid information access, centralized organization of clinical data, and checklist-guided documentation instead of exhaustive free-text recording.

#### 3.1.1 Step 1—CSD matrix

The construction of the CSD Matrix (Certainties, Suppositions, and Doubts) resulted in a total of 28 statements, organized into three categories: Certainties (*n* = 11), Suppositions (*n* = 11) and Doubts (*n* = 6). The full set of CSD Matrix statements (Certainties, Suppositions, and Doubts), including the complete list of items is provided in Supplementary Material S5.

Based on these initial premises, key design decision points were identified for the platform. Confirmation steps within the workflow and role clarity emerged as priorities, as core coordination tasks such as case selection, appointment confirmation, and in-round responsibilities were not consistently standardized across sites. Pre-round data availability and shared access also emerged as critical, as uncertainties persisted regarding information flow, including access to medical records and the sharing of new patient information with the Tele-ICU team. These findings pointed to the need for structured pre-round information-sharing routines and a shared, persistent tele-round record. Standardization with minimal burden was another key point, since workflows needed to remain predictable under time pressure without increasing documentation workload. Supporting the use of a checklist-guided round script and patient summary cards that translate decisions into explicit daily goals that persist beyond verbal discussion and can be exported or printed for hybrid workflows. Finally, embedded feedback and the capture of indicators were essential because monitoring, continuing education, and feedback mechanisms such as net promoter score (NPS), were positioned as part of routine operations. This implied integrating lightweight feedback capture and indicator-ready data structures into the workflow to support continuous monitoring and iterative improvement.

#### 3.1.2 Step 2-desk research

Building on the uncertainties identified in the CSD Matrix, desk research synthesized evidence to inform design priorities across five domains: engagement, feedback, infrastructure, information capture and sharing, and team composition. Nine peer-reviewed publications were selected (3, 6, 27–32), complemented by one book on engagement strategies and three technical sources describing tele-ICU hardware options (33–37); the full evidence-to-design mapping is provided in Supplementary Material S6.

Across sources, convergent findings emphasized that sustained tele-ICU adoption depends less on comprehensive documentation and more on trust-building and structured communication between hub and spoke teams, supported by audiovisual interaction and continued collaboration over time (6, 28, 32). The literature also highlighted the value of routine feedback loops and performance review to reinforce standardization and adherence (4). Feasibility constraints were recurrent, particularly connectivity instability and incomplete interoperability; thus, pragmatic solutions must prioritize rapid access and centralization of critical information even when full EMR integration is not available (28). Evidence comparing carts and tablets further illustrated trade-offs between technical robustness and scalability/cost in low-resource contexts (27). Finally, studies converged on the value of multidisciplinary tele-ICU teams anchored by intensivists, nurses, and physiotherapists, with flexible inclusion of other specialists depending on case complexity (31, 32).

These findings translated into four design and implementation priorities for Mangará Digital. First, the platform and operational workflow emphasized video-enabled, structured tele-rounds that support relationship-building through sustained collaboration rather than relying primarily on persuasive design elements (6, 28, 32). Second, a lightweight, repeatable feedback mechanism (net promoter score) was incorporated after rounds, complemented by periodic checkpoint meetings to support iterative improvement and governance (4). Third, given feasibility constraints, the project prioritized tablet-based deployment and avoided bandwidth-intensive features, while adopting a structured pre-round information-sharing routine to ensure availability of a minimum dataset (e.g., daily census and key exam results) (26, 28). Fourth, the system was designed to accommodate a core multidisciplinary team (physician, nurse, physiotherapist) with optional involvement of additional professionals when clinically indicated (31, 32), and it informed a longer-term direction toward shared visibility of treatment goals and multidisciplinary documentation (26).

#### 3.1.3 Step 3-rapid ethnography

Rapid ethnography highlighted both contextual differences and shared operational bottlenecks across a private and a public ICU (Table 1). Although both settings used digital systems, documentation practices differed substantially: the public ICU relied on hybrid records (paper-based, scanned, and electronic), whereas the private ICU used fully electronic medical records. Differences also emerged in performance monitoring routines: the private ICU followed a structured indicator collection schedule, while the public ICU lacked a defined monitoring agenda. Together, these observations indicated that the platform would need to remain functional under heterogeneous documentation modalities and variable levels of process standardization.

**Table 1:** Public vs. Private ICU Context: Comparative analysis and key bottlenecks affecting tele-rounds.

Domain/Theme	Contextual differences (Public VS. Private)	Bottlenecks
INFORMATION MANAGEMENT	Systems and medical records: • Private: Integrated ecosystem (TASY + EPIMED) with electronic medical records. • Public: Fragmented ecosystem (Magma + Hybrid records); partial reliance on paper and scanned documents.	Cognitive Load: Difficulty in locating critical data rapidly and information overload during rounds due to an excess of sources or fragmentation.
RITUALS AND ROUTINES	Preparation and monitoring: • Private: Established Pre-visit (round) routine and defined schedule for indicators. • Public: No pre-visit routine; lack of a defined monitoring schedule.	Constant Interruptions: Lack of prior alignment leads to frequent pauses to check connections, battery status, or basic pending items.
TEAM DYNAMICS	Checklist engagement: • Private: Active verbal check where everyone signs together. • Public: Passive task; Nurse registers alone using Google Forms.	Insecurity and Misalignment: Early-career physicians hesitate during complex decisions; verbal goals are often lost after the round ends.
VISUAL INFRASTRUCTURE	Type of ICU rooms: • Private: Single-bed rooms (20 beds). • Public: Multi-bed rooms (10 beds).	Spatial Disorientation: Difficulty in rapidly visualizing the global unit status and the location of critical patients.
HUMAN RESOURCES	Team composition: • Private: Intensive care physician, nurse, physiotherapist, speech therapist, psychologist, nutricionist • Public: Intensive care physician, nurse, physiotherapist, speech therapist	Role Overload: Information relevant to specialists can clutter the view for the core team, or vice-versa.

Beyond structural contrasts, ethnographic data revealed recurring workflow bottlenecks common to both environments. ICU professionals reported frequent interruptions, difficulty locating critical patient information during rounds, and clinical insecurity among early-career physicians during high-stakes decision-making. Nursing staff described work overload, limited recognition, and continued reliance on manual documentation, reinforcing the perception that documentation tasks compete with time-sensitive clinical work.

These findings supported prioritizing interface elements designed to reduce cognitive load and standardize multidisciplinary communication, including a checklist-guided tele-round structure, at-a-glance patient summary cards to minimize time spent retrieving information from multiple sources, and explicit daily goals to strengthen shared decision-making and continuity of care after rounds.

#### 3.1.4 Step 4-benchmarking

Benchmarking interviews with leaders involved in Brazilian Tele-ICU initiatives (Tele-ICU COVID and Tele-ICU Neonatal) highlighted how implementation planning and operational standardization shape program execution. Participants described a clear contrast between initiatives: the neonatal project was characterized by structured pre-planning and defined workflows, whereas the COVID initiative—implemented under emergency conditions—was reported as having limited upfront planning. This contrast was consistently associated with differences in operational consistency, including smoother coordination of activities, clearer role definition, and more predictable delivery of tele-round routines in the better planned initiative.

Across interviews, three enabling practices emerged as particularly relevant to Mangará Digital's design and deployment: (1) structured rounds supported by checklists to standardize case discussion and improve completeness of records; (2) use of an appropriate digital platform to organize documentation and facilitate indicator monitoring; and (3) a predefined agenda with fixed schedules, which was described as reducing absenteeism risk and improving team alignment.

These findings reinforced the need to embed standardization into both the interface and the operating model, prioritizing checklist-driven tele-round workflows, shared access to structured records and indicators, and scheduling/confirmation routines to sustain predictable execution across diverse ICU settings.

### 3.2 Define

Using personas, empathy maps, and situational diagnosis, the design team synthesized the findings into specific platform requirements to address user insecurities and environmental constraints.

#### 3.2.1 Step 5-personas and empathy maps

The personas and empathy maps (Supplementary Material S7) synthesized shared needs and constraints across ICU professional roles, translating them into concrete interface requirements. Analysis of these maps consolidated five critical pain points: (1) lack of time and cognitive overload; (2) fragmented access to clinical information; (3) absence of standardized workflows; (4) insecurity among young physicians during complex procedures; and (5) limited visibility of patient evolution and daily goals.

**Figure 2:**
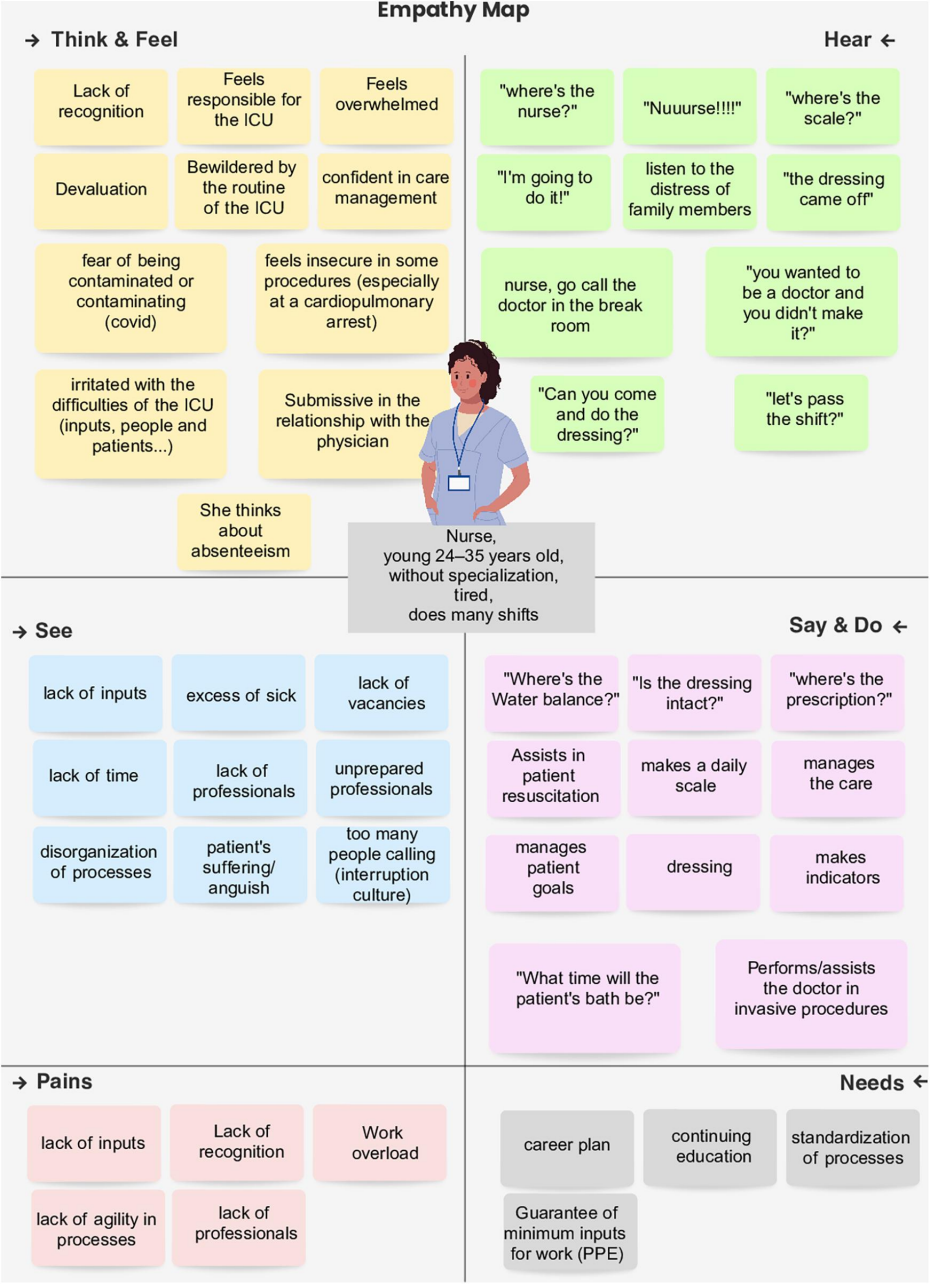
Persona and empathy Map of nurse persona.

These findings reframed the platform's usability objective: rather than adding another documentation layer, the system needed to support rapid shared sensemaking in an interruption-prone environment. The maps indicated that these constraints disproportionately affected early-career physicians, who reported clinical insecurity, and nurses, who often absorb the bulk of coordination and documentation work during rounds. Figure 2 illustrates the empathy map developed for the nursing professional persona, detailing their behavioral triggers, perceived gains, and pains.

Consequently, design priorities converged on a fast-access, low-navigation-burden interface. Specific usability features were prioritized to address these challenges: “patient summary cards” to reduce data retrieval friction, “standardized checklist-driven documentation” to stabilize multidisciplinary communication, and “explicit daily goal tracking” to make patient evolution readily interpretable.

#### 3.2.2 Step 6-situational diagnosis

Of 12 ICUs participating in the project, 10 (83.3%) responded. Data on infrastructure, workflows, and performance metrics were analyzed to classify ICU resources into three readiness levels, as summarized in Table 2, with the full results presented in Supplementary Material S8. This assessment revealed a clear dichotomy between domains: while clinical processes exhibited a high degree of maturity, the technological infrastructure remained critically fragile.

**Table 2:** Multidimensional readiness assessment of participating ICUs: strengths, areas for improvement, and bottlenecks.

Dimension	Strengths	Areas for improvement	Bottlenecks
	**≥90% of ICUs**	**60%–89% of ICUs**	**<60% of ICUs**
INFRASTRUCTURE & IT	Core services: Laboratory, X-ray, Surgical center Safety:Crash Cart (defibrillator and emergency medications) Equipment: Multiparametric Monitoring	Connectivity: Broadband and Wi-Fi in ICU Imaging: In-hospital, Bedside Ultrasound Renal: Intermittent Hemodialysis	Internet Stability: High frequency of outages (80% report outages) Digital Records: 0% fully electronic, 60% paper-based, and 40% hybrid Advanced Imaging: Magnetic Resonance Imaging Bedside echocardiography
CARE TEAM (Multidisciplinary Staff)	Team: Psychologists, Speech therapists, and Nutritionists Nursing: High density of nursing technicians (>15 per unit)	Pharmacist present in 80% of ICUs Physician availability consistent with unit size	
PROCESSES AND PROTOCOLS (Workflows and Safety)	Routines: Shift Handover, Daily Census, Multidisciplinary Rounds Safety Bundles: VAP, UTI, Sepsis, Hand Hygiene	Standardization: Continuous infusion drug solutions	
INDICATORS AND DATA (Monitoring Capacity)	Clinical: Incidence of ventilator-associated pneumonia (VAP) and urinary tract infection (UTI); mechanical ventilation (MV) utilization Outcome: ICU mortality rate	Operational: Length of Stay (ICU/Hospital) Device Use: Catheter utilization rates (CVC, Urinary)	Long-term Outcomes: 28-Day Mortality (Measured by only 40%)

First, a scenario of high process maturity (≥90% of ICUs) was identified. Critical care routines—such as shift handovers, daily census, and safety protocols (bundles)—were already institutionalized in nearly all units, alongside the presence of core multidisciplinary teams. This finding was pivotal for the design strategy: rather than introducing new workflows, the platform was designed to mirror and operationalize these existing rituals through embedded checklists and role-based task cues. This approach enabled digital continuity without forcing teams to reinvent routines, thereby reducing the learning curve.

In contrast, the digital infrastructure presented severe bottlenecks. Although nominal broadband availability fell within the Intermediate Level, effective connection stability proved to be a Critical point (<60%), characterized by frequent outages occurring at least 2–4 times per month. This fragility necessitated a resilient, offline-first architecture featuring a lightweight interface, autosave capabilities, and graceful degradation to prevent workflow disruption during tele-rounds. Concurrently, documentation practices were heterogeneous, with a substantial proportion of units relying on paper-based or hybrid medical records. This reinforced the need for flexible data capture that functions without EMR integration, prioritizing structured fields over free text to centralize key information and prevent fragmentation.

Furthermore, the diagnosis exposed a strategic gap in performance indicator management. While immediate metrics (such as general mortality and length of stay) were widely monitored (≥90% of ICU), justifying the integration of dashboards and trend visualizations for shared situational awareness and performance feedback, long-term outcomes remained at a critical monitoring level.

#### 3.2.3 Step 7-user journey mapping

**Figure 3:**
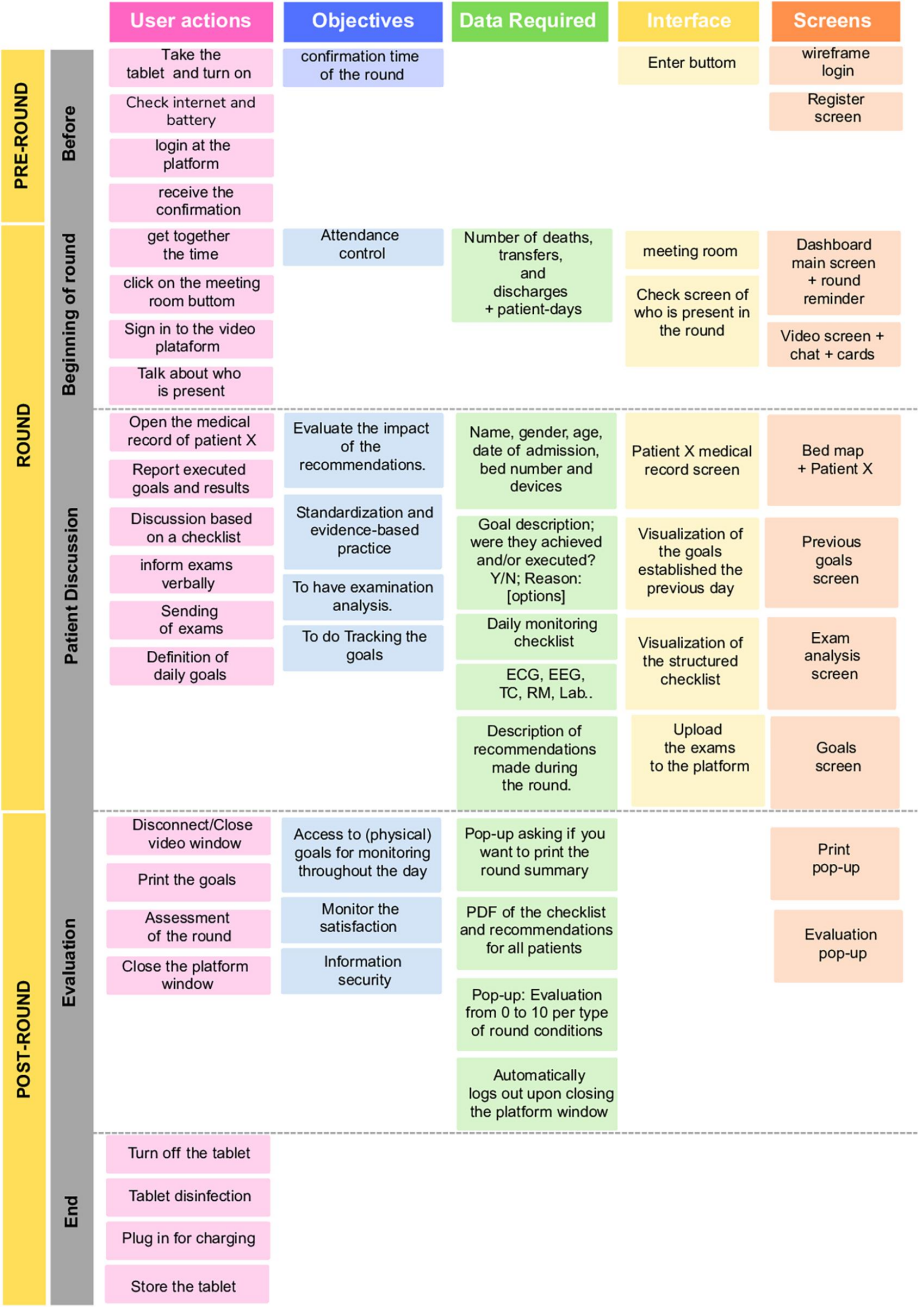
User journey.

The user journey included three main stages: pre-round, round, and post-round. Figure 3 presents this journey, highlighting the user's interactions with the system, the clinical and operational objectives at each stage, and the corresponding interface and screen components.

In the pre-round phase, key actions included confirming the tele-round schedule, verifying the tablet's battery and internet connection, logging into the platform, and joining the virtual meeting room. The system was designed to support the organization of this process through automated reminders and participant verification, aiming to reduce delays and ensure a structured start to clinical discussions.

During the tele-round, the care team accessed the patient's digital record directly through the platform. Discussions were guided by structured checklists, which supported standardized clinical evaluations. The team reviewed the goals established the previous day, shared recent exam results (either verbally or via file upload), and collaboratively defined new care objectives. This phase required a fast, intuitive interface that facilitated access to essential clinical data, promoting safety, traceability, and consistency in decision-making.

In the post-round phase, users assessed the quality of the tele-round using a 0–10 rating scale (Net Promoter Score—NPS). The platform also enabled users to print or save a summary of the goals and clinical recommendations, facilitating continuity of care and daily goal tracking.

Journey mapping revealed distinct friction points across phases. Before rounds, uncertainty about scheduling and dispersed patient data increased anxiety and absenteeism risk. During rounds, lack of shared visual structure led to interruptions and prolonged discussions. After rounds, goals were frequently lost in verbal communication, impairing execution and monitoring. These bottlenecks directly informed the inclusion of confirmation mechanisms, integrated video–checklist views, and visualization of daily goals.

#### 3.2.4 Step 8-sitemap and userflow

Building on the evidence synthesized in the Discover and Define phases, user needs and workflow requirements were translated into the platform's information architecture and interaction structure. To minimize navigation burden and cognitive load, the sitemap followed a shallow hierarchy and prioritized direct access to the core modules. Key areas such as the indicator entry and visualization area, ICU bed mapping, the patient list, and the medical record were reachable in few steps. A central design decision was to adopt a patient “card” landing screen that summarizes essential information at a glance, informed by usability principles and a evaluation based on Jakob Nielsen's heuristics (Supplementary Material S9).

In parallel, a user flow was created to operationalize a representative clinical scenario and verify alignment between the interface structure and real ICU work. The flow mapped the interactions required for a bedside nurse to register a new patient, participate in a multidisciplinary tele-round, share diagnostic exams, and retrieve or print daily goals after the round. This “before, during, and after” journey ensured that the architecture supported time-critical decision-making and continuity of care. Early wireframes for the tele-round documentation screen were developed from these artifacts, and implementation began after this ideation and prototyping step. The complete sitemap and user flow diagrams are presented in Supplementary Material S10 and S11.

#### 3.2.5 Step 9-wireframes construction

The wireframe construction phase translated the information architecture into high-fidelity prototypes. To directly address the cognitive overload identified in the Discover phase, the interface prioritized a strong visual hierarchy.

**Figure 4:**
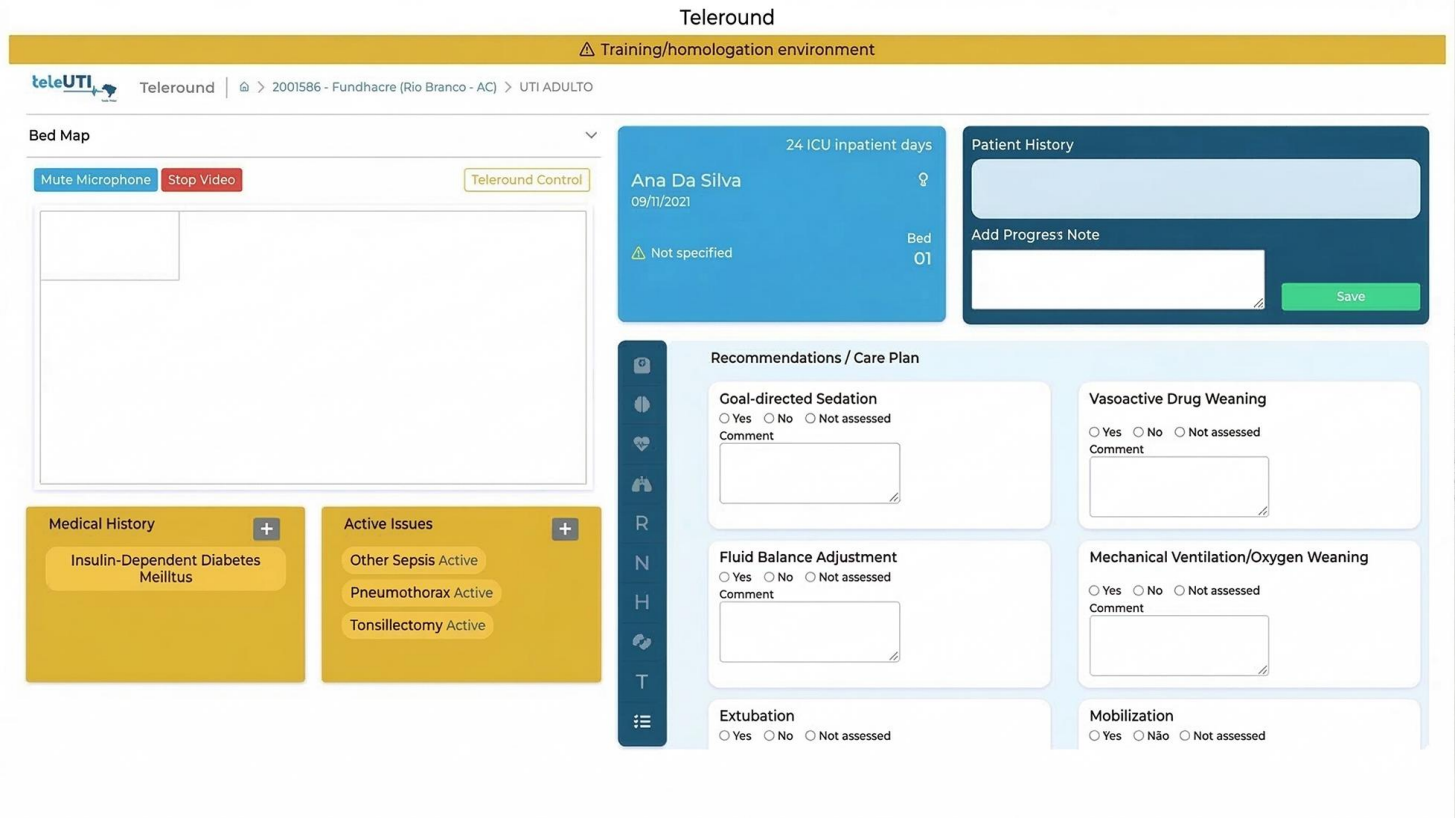
High-fidelity screen of the tele-round platform (data does not include the details of any real patient).

Patient summary cards were incorporated to display key information at a glance, including age, name, length of stay, and known allergies. These cards were intended to provide a concise and easily interpretable overview of essential patient data (Figure 4).

Furthermore, the initial screen provides the healthcare team with a comprehensive map of the ICU's bed layout, facilitating efficient bed management and patient assignment. This visual representation enables informed decision-making regarding patient allocation and resource utilization.

**Figure 5:**
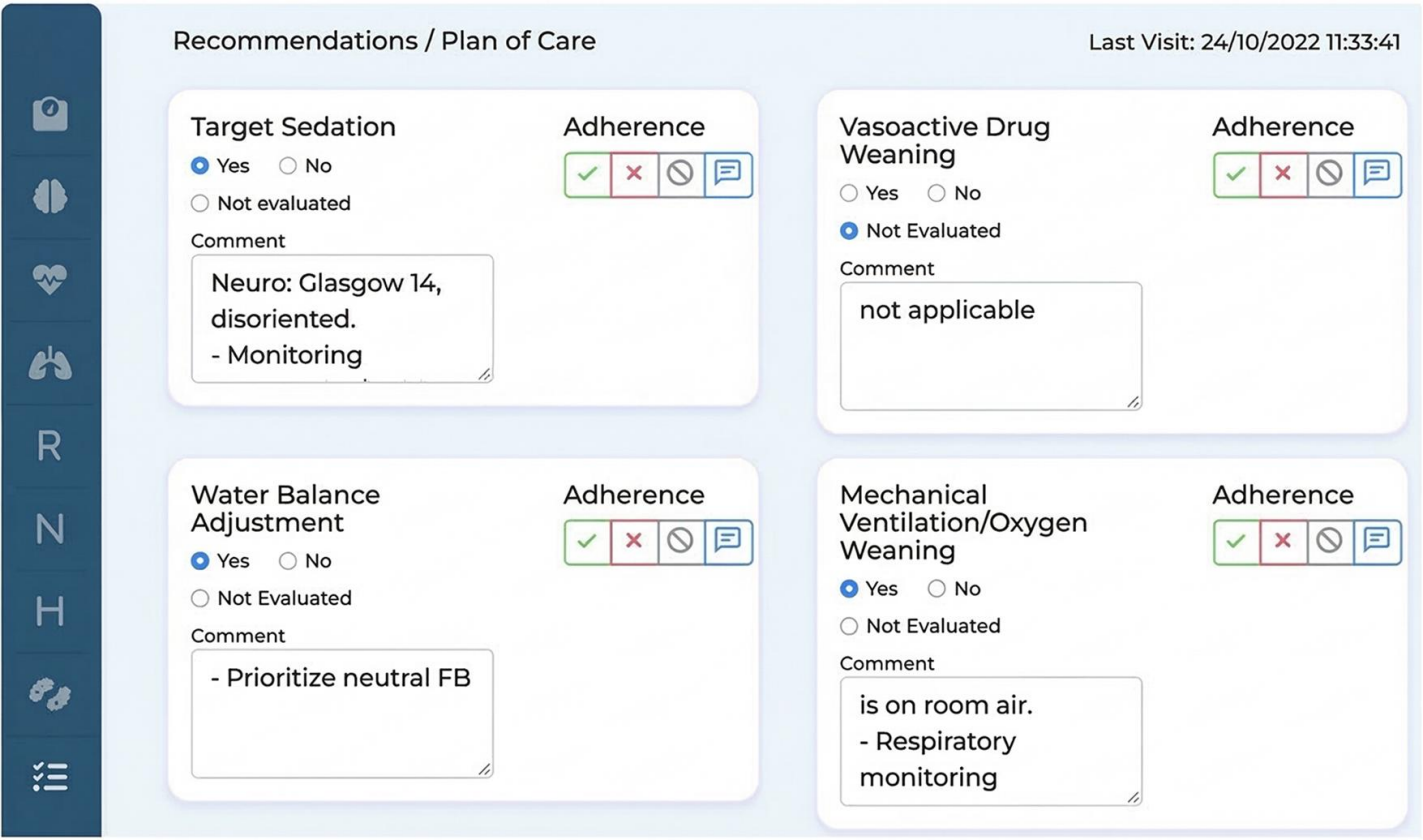
Screen to record adherence to tele-round recommendations (data does not include the details of any real patient).

Additionally, the rounding screen offers a wealth of vital information for healthcare professionals. It presents the patient's problem list, offering insights into the patient's specific health issues. Moreover, it provides:

1. A comprehensive compilation of all the necessary data gathered from the daily checklist.
2. A comprehensive overview of the patient's condition.
3. Treatment progress.
4. A structured space to document recommendations, care plan items, and adherence to tele-round decisions. Figure 5 presents the tele-round documentation screen used to record adherence to recommendations made during the tele-round.

#### 3.2.6 Step 10-testing and validation

The final phase focused on interface validation through heuristic evaluation and continuous post-launch monitoring. This real-world validation reinforced the core assumptions identified during the Discover phase, particularly the critical need for lightweight interfaces, clear navigation, and tolerance to low connectivity.

Post-launch feedback revealed specific usability frictions that guided the platform's iterative refinement. For instance, users experienced initial challenges with the database during the registration process and difficulties customizing specific bed information within the visual map. More significantly, frequent audio and video instabilities in low-bandwidth regions hindered live clinical communication. These insights directly drove the implementation of an asynchronous chat feature, allowing the team to maintain communication via text and image sharing even when video failed.

## 4 Discussion

Developing digital systems for critical care environments such as intensive care units is inherently challenging because clinical work is time-sensitive, interruption-driven, and high risk, while infrastructure and documentation practices often vary substantially across sites (38–40). This study demonstrates how a structured user experience process can translate field constraints into an implementable tele-round platform under real-world conditions. Using the Double Diamond methodology, the Mangará Digital project progressed from identifying user needs and infrastructure limitations to defining requirements and delivering interface functionalities that supported multidisciplinary tele-rounds in routine practice. These findings reinforce a central premise in digital health implementation: human-centered design is not only a development strategy, but also a risk-mitigation approach that enhances the likelihood of adoption and sustained use in complex clinical systems.

Mangará Digital was deployed across 15 public ICUs (went live on June 21, 2022) and supported 3,971 tele-rounds for 5,471 distinct patients through November 30, 2023, indicating feasibility at scale and sustained use in routine clinical work (20). Implementation indicators further support operational feasibility. During the first 18 months of operation, the program recorded 20,168 telehealth encounters, with a median duration of 9 min 42 s (20). Most patients received at least one telehealth encounter per day (74.0%). Among cases discussed during tele-rounds, bedside teams adhered to 97% of daily goals, and professional satisfaction was very high (NPS 97.96) (20). Taken together, these findings support the interpretation that the platform functioned as a care coordination instrument by structuring clinical communication, reducing information loss, and helping sustain continuity after the synchronous encounter.

Across rapid ethnography, situational diagnosis, empathy mapping, and journey mapping, five constraints emerged: severe time pressure; fragmented access to clinical information; limited workflow standardization; uncertainty among less experienced professionals during high-stakes decisions; and limited visibility of patient evolution and daily goals. These constraints were translated into three core UX mechanisms that help explain the platform's practical value in this context. First, speed-oriented navigation reduced interaction costs by enabling immediate access to essential patient information through concise screens and shallow information architecture. Second, checklist-guided documentation reduced cognitive load and strengthened multidisciplinary communication by structuring discussion content and decreasing reliance on free-text entry. Third, persistent and exportable daily goals supported continuity by ensuring that round decisions remained visible and actionable for spoke teams. Interpreting Mangará Digital through these mechanisms clarifies that its primary contribution is enabling shared cognition and workflow coordination during tele-rounds, rather than merely digitizing documentation.

Post-launch monitoring further demonstrates the importance of designing for real-world constraints and enabling iterative refinement. Early feedback identified friction in user registration and challenges in customizing bed-level information in the ICU map, which prompted targeted interface refinements to reduce interaction burden and improve unit-level orientation. More critically, recurrent audio and video instability in low-bandwidth settings hindered synchronous communication; in response, an asynchronous chat function was implemented to preserve clinical dialogue through text and image sharing when video failed. These adaptations illustrate two design lessons that are especially relevant in critical care telehealth: usability requirements continue to evolve after deployment, and resilience features (including the ability to maintain core functions under degraded connectivity conditions and the use of asynchronous alternatives) can be as important as primary “happy-path” interaction flows.

When considered alongside the literature, these findings reinforce evidence that UX-driven telehealth design improves usability and adoption, particularly when aligned with real clinical workflows (41). Similar benefits of Double Diamond and participatory approaches have been reported in other telehealth contexts (8, 9); however, this study extends prior work by demonstrating how these methods can be applied in Brazilian public ICUs characterized by resource constraints and infrastructural heterogeneity. Importantly, the design process shifted the platform's value proposition from documentation toward supporting clinical reasoning, workplace learning, and visibility of daily goals. This repositioning is particularly relevant in high-pressure environments, where the interfaces must reduce cognitive friction and maximize alignment across teams.

Relative to participatory design and co-creation frameworks, in which users act as continuous co-designers and contribute generative artifacts throughout the process, our engagement model was necessarily pragmatic (42). Clinical workload, multi-site logistics, and patient-safety priorities limited the feasibility of sustained co-design workshops. Accordingly, clinician participation occurred primarily via focused interviews, rapid feedback cycles on prototypes, and iterative refinements after deployment. A deeper adoption of participatory design might have positioned health professionals as more direct co-authors of the interface, potentially enabling earlier detection of workflow barriers and reducing the need for reactive post-launch adjustments. At the same time, the time and resource demands of intensive co-design often conflict with operational realities in critical care. This supports the rationale for a situated human-centered design approach that remains implementation-oriented while still preserving meaningful user input. Future iterations could incorporate participatory mechanisms that remain clinically feasible, such as rotating clinician-designers among clinicians, short profile-specific co-design sprints (nursing, physicians, physiotherapy), asynchronous storyboarding, and brief periodic remote workshops to broaden representativeness without disrupting care delivery.

From a usability engineering perspective, this study operationalized core human-centered design activities consistent with ISO 9241-210 (43): understanding the context of use, specifying user requirements, producing design solutions, and iteratively evaluating designs. The Discover phase generated evidence about the context of use through observations and benchmarking; the Define phase translated insights into explicit requirements and an initial information architecture; the Develop phase operationalized requirements through prototyping and iterative validation; and the Deliver phase emphasized deployment and monitoring in routine practice. This mapping positions the work as human-centered design executed under real implementation constraints, rather than as a descriptive account of design stages.

A key methodological consideration concerns evaluation. Formal pre-deployment usability testing using standardized task-based measures was not feasible within the project timeline. Instead, the team relied on heuristic review, prototype walkthrough sessions, and structured post-launch feedback cycles, including routine monitoring mechanisms such as NPS. This strategy increases ecological validity by capturing usability under authentic workflow conditions, but it may delay the identification of specific interaction issues that controlled testing can detect earlier. Accordingly, the evidence should be interpreted as a field-validated rather than a laboratory-validated pathway, supporting future studies that combine real-world deployment with standardized usability, safety, and performance outcomes.

The deployment context in Northern and Northeastern Brazil is essential for interpretation. Barriers include unstable connectivity and power supply, limited availability of local IT support for system configuration and maintenance, variable device replacement cycles, hybrid documentation ecosystems (paper, scanned, and partial electronic records), and organizational constraints such as high turnover and limited availability of locally trained intensivists. These socio-technical realities shape both usability and adoption: teams must retrieve and reconcile fragmented information under time pressure; digital literacy and confidence can vary across sites and professional categories; and trust-building can be necessary when remote support is initially perceived as external oversight rather than collaborative care. To improve operational resilience under intermittent connectivity, the program prioritized a lightweight interaction model and mitigation strategies, including 4G data plans, mobile devices, and wireless routers. Capacity building was also central, with multimodal educational strategies (in-person workshops, recurring virtual learning sessions, and simulation-based training) supporting adoption and confidence development. High satisfaction metrics further suggest that positioning the platform as a tool that structures dialogue and makes daily goals visible, rather than as an additional documentation burden, is central to acceptance in settings with variable resources. The post-launch inclusion of asynchronous chat is an example of how designing explicitly for socio-technical constraints can strengthen operational robustness when synchronous telepresence is intermittently compromised.

Framing this work as applied design science further strengthens its contribution to digital health and human-computer interaction. The primary output is a socio-technical artifact intentionally built to address a field problem and evaluated through use in its implementation context. This aligns with design science research (44) and action design research (45), which emphasize iterative intervention in context and the abstraction of reusable principles. From this perspective, the Double Diamond activities function not only as a development narrative, but also as a mechanism to derive empirically grounded design principles for ICU telehealth documentation and coordination.

Across phases, findings support transferable UX principles for tele-round support in high-risk environments (1): minimizing interaction costs under time pressure through speed-oriented navigation and concise screens; (2) reducing cognitive load via structured capture (checklists and standardized fields) rather than reliance on free text; (3) preserving continuity of care by keeping goals visible, persistent, and exportable for spoke teams; (4) designing for low bandwidth and operational resilience through lightweight interfaces and contingency pathways; and (5) supporting coordination before and after synchronous rounds, including participant verification, reminders, and post-round summaries. These principles translate observed constraints into actionable guidance for future Tele-ICU and teleconsultation platforms and help explain how Mangará Digital addressed barriers frequently described in telehealth implementations, including fragmented information flows, lack of standardized workflows, and the need for reliable communication tools in constrained environments (1–5, 13, 16, 17).

This study has limitations. First, the absence of formal pre-deployment usability testing with standardized measures may have delayed identification of specific interaction issues, although iterative post-launch feedback enabled corrective cycles under real workflow conditions. Second, ethnographic observations were concentrated in a limited number of ICUs located in a more developed context (São Paulo), which may limit transferability to services with different infrastructure and organizational maturity; however, this risk was partially mitigated by triangulation with the situational diagnosis across participating services and benchmarking with other Tele-ICU initiatives. Third, design research often relies on small samples studied in depth, which increases susceptibility to selection bias when not all professional profiles can participate and some groups may be under-represented. Additional research across other regions, with broader professional representation and complementary participatory strategies, is needed to test the robustness of the proposed principles and to quantify their effects on usability, safety, and clinical performance outcomes.

Despite these limitations, our findings indicate that combining the Double Diamond framework with a pragmatic human-centered design approach can yield an implementable platform to support tele-rounds in high-stress ICU contexts with variable resources. By explicitly linking field constraints to design responses and by documenting how resilience features emerged through deployment, this study contributes transferable knowledge for telehealth tools that must operate under constraints related to infrastructure, budget, time, and workforce, and it offers actionable principles to guide the development of future Tele-ICU solutions.

## Data Availability

The raw data supporting the conclusions of this article will be made available by the authors, without undue reservation.
