# Supplementary material for: User experience design methodologies for developing a tele-round platform in public intensive care units in northern and northeastern Brazil
Source: Front Digit Health. 2026 Apr 8;8:1713349. doi: 10.3389/fdgth.2026.1713349 (PMC13099869; doi:10.3389/fdgth.2026.1713349)
Supplement: Supplementary file 1 [file Supplementaryfile1.docx]

**Supplementary material 1. Overview of the double diamond stages and their main outcomes**

| **Stages** | **Steps** | **What ??** | **When ?** | **Participants** | **Data Collection** | **Data Organization** | **Data Analysis** | **Outputs/ Outcomes** |
| --- | --- | --- | --- | --- | --- | --- | --- | --- |
| **DISCOVER** | CSD Matrix (Certainties, Suppositons and Doubts) | 1 meeting (2h) |  | Members of design teams | Brainstorming | The data collected was recorded on the Miro platform | The data collected was classified into certainty, assumption or doubt about the project and its context. | Understanding the universe of the problem. |
|  | Desk Research (DR) | Literature review +  2 synchronous meetings |  | Members of design teams | Literature review in databases of scientific publications and websites    +  Group discussions  on the results identified in the literature. | Data was be recorded in the Excel platform and structured in the Power Point program for further discussion of the results. | Content analysis was performed. The results of the studies were categorized according to the following topics: methods of engagement, user perception, technological structure, communication strategies, multidisciplinary team, data collection and sharing.. | -Understanding of the main problems in digital health in Brazil and in the world.  -Mapping about project experiences and technological solutions, whether in the health area or not, in Brazil and/or abroad, in order to generate insights that can help decision-making. |
|  | Rapid Ethnographic Research | Face-to-face visits to an ICU of a private hospital (5 hours) | March 2022 | -UX Designer (researcher)  ]  -Private ICU staff members:  3 intensivist physicians,  2 nutritionists,  2 physiotherapists,  3 nurses.  2 residents and  1 psychologist). | Semi-structured interviews    +    Participant  observation | The collected data were registered manually in a physical form. | A comparative analysis of the ICUs was carried out in order to guarantee a better perspective of the daily life of an ICU and to build the best experience. | Bringing the design team closer to the reality and processes in the ICU, as well as the behavioral patterns of the multidisciplinary team in the scenario, understanding the main triggers and insights of the users, allowing the construction of a journey that offers the best possible experience. |
|  |  | Face-to-face visits to  an ICU of a public hospital in a region with low economic conditions (5 hours) | March 2022 | UX Designer (researcher)  - Public ICU staff members:  1 Platonist doctor  1 speech therapist  1 physiotherapists  1 nurses  2 nursing technicians |  |  |  |  |

**Supplementary material 1. Overview of the double diamond stages and their main outcomes**

|  | **Steps** | **What ??** | **When ?** | **Participants** | **Data Collection** | **Data Organization** | **Data Analysis** | **Outputs/ Outcomes** |
| --- | --- | --- | --- | --- | --- | --- | --- | --- |
|  | Benchmarking | Remote interviews | March 2022 | - Members of design team  -Coordinator of \|Neonatal Tele-ICU project  -Coordinator of Covid Tele-ICU project | Semi-structured interviews | The collected data were recorded on the Notion platform | Comparative Analysis of project practices and experiences (in their different structures and scenarios) seeking points of convergence with the scope of the project and feasibility of adaptation and execution. | Mapping the structure, execution and processes of similar digital health projects in order to understand the main barriers and facilities to support decision-making with a view to the best results and strategies. |
| DEFINE | Personas e Mapa de Empatia | Remote meeting (2hours) | April 2022 | Members of design team | Group discussions + brainstorming | The collected data were recorded on the Miro platform | The empathy map was built based on the survey of information in the previous stages, that is, in this stage the information has already been analyzed and will be organized | Understanding the context and behavioral aspects of physicians and nurses who work in public ICUs |
|  | Situational Diagnosis | Questionnaires via the Survey platform |  | Coordinators of the ICUs that are part of the project | self-administered questionnaire | The collected data were recorded on the surveys platform.. | A descriptive analysis of the answers obtained in the forms was carried out. A comparative analysis of the data was also carried out with the aim of identifying differences in the resources available in each ICU.  Finally, an analysis of the main initial barriers that could impact the execution and effectiveness of the project was carried out. | Knowing the structure and resources available in the ICUs, identifying problems and needs in order to anticipate predictions and solutions.      Building a user journey that can be applied in different scenarios (knowing their common patterns and difficulties). |

| **Supplementary material 1. Overview of the double diamond stages and their main outcomes** | | | | | | | | |
| --- | --- | --- | --- | --- | --- | --- | --- | --- |
| **Stages** | **Steps** | **What ??** | **When ?** | **Participants** | **Data Collection** | **Data Organization** | **Data Analysis** | **Outputs/ Outcomes** |
| DEVELOP | User Journey | Construction of the initial journey (2 synchronous meeting) | April 2022 | Members of design team | Group discussions, Brainstorming | The collected data were recorded on the Miro platform | The User's Journey was built based on the collection of information in the previous stages, that is, in this stage the information has already been analyzed and is organized in a structured way.  For data structuring, a map was systematized in vertical columns addressing the following topics: patient entry, ICU routines, case discussions | Architect the user journey of the TeleUTI project considering the assumptions and knowledge acquired in the previous steps. |
|  |  | Detailed Journey Construction -6 synchronous meetings (1-3 hours) | April 2022 | Members of design team  + Stakeholders:  Digital Health Project Coordinators, Developer, Data Specialist |  |  |  |  |
|  | Userflow + Sitemap | Creation of userflows and sitemap | April 2022 | UX designer | ----------------- | Mapping of the main tasks and workflows that users perform on the product using the Figma Platform. | Hierarchical analysis of tasks, specifically for the purpose of developing our prototype. | Visual representation of tasks and paths users can take when interacting with the product |
|  | construction “Wireframe” | Meetings | April 2022 | UX/UI designer (responsible for building the architecture in the tool), a project specialist expert in data and digital health and an expert in health intelligence. | Group discussions, Brainstorming | Figma platform | Systematization of the feedback obtained. | Visually structure the organization of  platform elements and validate the feasibility of the planning defined in the previous steps |
| DELIVER  ( Test + Validation) | Testing and Validation of the User flow and the  wireframes | Meetings | May 2022 | UX Designer + TeleICU Project multidisciplinary team (doctors, nurses, physiotherapists) | - Group discussions | The collected data will be registered on the Figma platform by the UX Designer | - Systematization of feedback obtained with the aim of identifying opportunities for improvement. | Discuss screen views and technical and functional details |
