# Supplementary material for: User experience design methodologies for developing a tele-round platform in public intensive care units in northern and northeastern Brazil
Source: Front Digit Health. 2026 Apr 8;8:1713349. doi: 10.3389/fdgth.2026.1713349 (PMC13099869; doi:10.3389/fdgth.2026.1713349)
Supplement: Supplementary file 2 [file Supplementaryfile2.docx]

**Supplementary material 2. Semistructured interview guide**

**CONTEXT**

1. How many beds are there in the ICU?

2. Is there a semi-intensive care service? Is it common for there to be semi-intensive patients in the ICU?

3. How is the preparation for the rounds?

4. How can you access patient data and where is it stored? (Examples of data sources include EPIMED, medical records, and the hospital system.)

5. Is there a method to digitize the patient data?

**HUMAN RESOURCES**

1. Which professionals comprise the multidisciplinary team in the intensive care unit? Who are the members?

2. What is the nursing routine in the ICU? What activities are included in the nursing routine within the ICU?

3. What are the routines for nursing technicians, physiotherapists, and nutritionists?

4. Are the doctors who are part of the ICU team on duty or permanent?

**PROCESSES**

1. Which processes are digitalized, and which are completely manual? Examples: Medical records, Medical evolution, Prescription of medicines and exams.

2. Are the mandatory indicators required by ANVISA collected in the ICU?

3. How is data collected? Is there specialized software for this purpose?

4. Who is responsible for recording these indicators and gathering this data?

5. Is there a certain amount of records they fill out on a daily basis?

6. Are the exams in digital format or are they provided as printed documents?

7. Could you explain how the record-keeping during rounds operates? Who is responsible for the documentation? Is there a digital system in place for this purpose?

8. Is there a standardized checklist used, or is the documentation more of a free-form text produced by the team?

9. What are the Standard Operating Procedures used in the Intensive care unit?

10. How the multidisciplinary team relationship works?
