# Supplementary material for: User experience design methodologies for developing a tele-round platform in public intensive care units in northern and northeastern Brazil
Source: Front Digit Health. 2026 Apr 8;8:1713349. doi: 10.3389/fdgth.2026.1713349 (PMC13099869; doi:10.3389/fdgth.2026.1713349)
Supplement: Supplementary file 3 [file Supplementaryfile3.docx]

**Supplementary material 3**

**Semistructured interview guide with project managers involved in implementing telerounds within Brazilian public ICUs (benchmarking).**

STRUCTURE

1. What type of device is employed for telerounds? [video]

2. What are the main obstacles encountered in the situational diagnosis of ICUs ?

3. What main changes were necessary [platform]?

4. Is there currently a pain point that professionals always point out in the structure of the project?

PROCESSES

1. How can we encourage teams to participate in telerounds and maintain their involvement actively?

2. Is there an assessment process for each teleround?

3 How do ICU teams access project records?

4. Do the teams use a checklist standardized by the project?]

5. When was the schedule for the telerounds agreed upon [during implementation]?

6. How effective is the scheduling flexibility, and what impact does it have on the overall operation?

7. What was the procedure for receiving exams?

8. Is there Agenda management?

9. Is a designated person responsible for managing requests and issues such as remote tips, support, and round delay messages?

10. Who was responsible for the scheduling of telerounds?

12. Is there a location-based agenda in place?

HUMAN RESOURCES

1. Who collects the daily indicators?

2. What essential professional categories are indispensable for telerounds?

3. Who is responsible for agenda management? How many nurses are currently involved in the project? What is their role in the project?

4. Can you specify which members are involved in the teleround?
