# Supplementary material for: User experience design methodologies for developing a tele-round platform in public intensive care units in northern and northeastern Brazil
Source: Front Digit Health. 2026 Apr 8;8:1713349. doi: 10.3389/fdgth.2026.1713349 (PMC13099869; doi:10.3389/fdgth.2026.1713349)
Supplement: Supplementary file 4 [file Supplementaryfile4.docx]

**Supplementary material 4**

**Questionnaire developed by the authors for situational diagnosis**

**GENERAL INFORMATION ABOUT THE HOSPITAL**

1. Hospital name:

2. Status:

3. Name of person responsible (for filling out):

4. Reference Contact:

5. Email:

6. Date of filling out the form:

7. Number of beds:

8. Do you have a surgical center?

9. Exams carried out at the hospital:

( ) Laboratories If not, where:

( ) X-ray, If not, where:

( ) Magnetic Resonance, If not, where:

( ) Computed tomography, If not, where:

( ) Ultrasound, If not, where:

( ) Echocardiogram, If not, where:

( ) others Which:

10. Do you have interventional radiology (cardiac catheterization)?

11. Do you have interventional radiology (neuro)?

12. Do you have interventional radiology (general)?

13. Do you have dialysis equipment (intermittent method; continuous method)?

14. Are there institutional protocols for the ICU made by Hospital Infection Control Commission?

**INFRASTRUCTURE OF THE ICU**

1. How many beds?

2. Did they respond to COVID? If yes: From ____ Until______

3. Do you use electronic or physical medical records or both?

4. Do you have broadband internet or Wi-Fi?

5. Enough area for the movement of people and carts?

6. Do you have isolation beds?

7. Do you have wall-mounted breathing assistance?

8. Do you perform bedside X-rays? If not, how do you transport it? (internal and external)

9. Do you perform Ultrasound in the unit? If not, how do you transport it? (internal and external)

10. Do you perform a bedside Echocardiogram? If not, how do you transport it? (internal and external)

11. Who transports the patient outside the ICU? (internal and external)

**HUMAN AND MATERIAL RESOURCES**

1. The ICU team is made up of:

( ) Doctor, How many?

( ) Nurse, How many?

( ) Physiotherapist, How many?

( ) Nutritionist How many:

( ) Speech therapist How many?

( ) Others: Which?

2. Do you have intensive care doctors?

3. Do you have intensive care nurses?

4. Do you have an intensive care unit?

5. Professionals with a permanent contract? How many?

6. Professionals with a temporary contract? How many?

7. PJ professional in the ICU?

8.Weekly workload (for each professional category):

Is there a routine doctor or exclusive on-call doctor?

Is there a routine nurse?

Is there a routine physiotherapist?

12. Do you have multiparametric monitoring?

13. How many pulmonary ventilation equipment units are available in the unit?

24. Do you have a kit (“stop cart”) with defibrillator/cardioverter with medicines and emergency materials?

**WORKFLOWS**

1. Which medicines are standardized?

2. Do you use an instrument/form for shift handovers?

3. Does the nursing team use the Nursing Care Systematization (SAE)?

4. Do you carry out a multidisciplinary round/visit? What time?

5. Do you use a bundle to prevent primary bloodstream infection?

6. Do you use a bundle to prevent urinary tract infection?

7. Do you use a bundle to prevent pneumonia associated with mechanical ventilation?

8. Do you carry out daily census collection? If yes: who does it? At what time? Where (do they use epimed?)?

9. Do you perform Ultrasound US-guided CVC puncture?

**ICU INDICATORS**

The following indicators are monitored:

1. Incidence density of pneumonia associated with mechanical ventilation

2. Catheter-associated bloodstream infection density

3. Density of urinary tract infection associated with bladder catheter

4. Central Venous Catheter (CVC) utilization rate

5. Urinary catheter utilization rate

6. Mechanical-Ventilation (MV) utilization rate

7. Pressure injury incidence

8. Tracheostomy utilization rate

9. ICU mortality rate

10. Mortality rate (D28)

11. ICU length of stay

12. Length of hospital stay
