# Supplementary material for: User experience design methodologies for developing a tele-round platform in public intensive care units in northern and northeastern Brazil
Source: Front Digit Health. 2026 Apr 8;8:1713349. doi: 10.3389/fdgth.2026.1713349 (PMC13099869; doi:10.3389/fdgth.2026.1713349)
Supplement: Supplementary file 5 [file Supplementaryfile5.docx]

Supplementary Material 5. CSD Matrix statements and categorization.

The construction of the CSD Matrix (Certainties, Suppositions, and Doubts) resulted in a total of 28 statements, organized into three categories:

1. Certainties (n = 11):

- Daily meetings with local teams lasting one hour each, led by the same physician assigned to each ICU;
- A Tele-ICU Project team composed of three physicians, two nurses, and two physiotherapists;
- Use of video for conducting tele-rounds;
- Maintenance of a structured tele-round record;
- Register of tele-round available for both teams;
- Informed Consent Form;
- Monitoring of clinical and operational indicators;
- Implementation of continuing education activities;
- Incorporation of feedback mechanisms such as the Net Promoter Score (NPS);
- Flexibility in scheduling to accommodate the operational dynamics of each ICU.
- Key indicators: Duration of mechanical ventilation; Length of ICU stay; Turnover of professionals in ICUs duration of tele-rounds; Participation in training activities; Number of telehealth visits (beds) / day.

1. Suppositions (n = 11):

- Teleconsultants will form a multiprofessional team (including nurses and physiotherapists);
- Patient registration forms will be sent to the participating ICUs;
- Evaluation of the outcome of each patient;
- Semi-intensive patients will not be discussed;
- Application of the Inform consent form will be according to the routine of the unit: manual and/or digital;
- General weekly evaluation of tele-rounds;
- It is necessary to manage appointments (confirmation);
- The visit of the local doctor must be standardized (established method);
- Making scheduling more flexible can increase the risk of absenteeism;
- The pre-visit and availability of information can facilitate the dynamics of the tele-round.

3.Doubts (n = 6):

- Who is responsible for selecting the patients to be discussed during tele-rounds?
- What should the structure and script of the tele-rounds look like?,
- Could we conduct a discovery session with professionals who have previously participated in a Tele-ICU initiatives?
- How should appointment confirmations be managed (e.g., by phone, chat, or WhatsApp)?
- How will the ICU team access project records?
- How will new patient information be shared with the Tele-ICU team?
