# Supplementary material for: User experience design methodologies for developing a tele-round platform in public intensive care units in northern and northeastern Brazil
Source: Front Digit Health. 2026 Apr 8;8:1713349. doi: 10.3389/fdgth.2026.1713349 (PMC13099869; doi:10.3389/fdgth.2026.1713349)
Supplement: Supplementary file 6 [file Supplementaryfile6.docx]

| **Supplementary material 6. Description of selected studies in desk research** | | | | | | |
| --- | --- | --- | --- | --- | --- | --- |
| **Topics** | **Title** | **Year** | **Context** | **Solutions/Insigts identified in the literature review** | **Solutions/ Insights identified in the Ideation process** | **Solutions Implemented** |
| Engagement | Identification of tele-ICU system requirements using a content validity assessment    Designing a critical care solution using in-person and telemedicine approaches in the US-Mexico border area during COVID-19    Hooked: How to Build Habit-Forming Products | 2016  2021 | European intensive care professionals  Community hospitals from United States (US) and Mexico | - ICU physicians should be involved from the beginning of the tele-ICU design and implementation.    - Physicians from the monitoring center should visit the teams at the bedside in order to build trust on both sides of the camera.    - Audio-visual contact with the tele-consultant is better than just audio contact. | Engagement would be accomplished by building habits and nudges within the platform. We also thought about presenting a gamification project within the platform. | The engagement came from building an interpersonal relationship during the deployments, and sustaining the relationship created throughout the telerounds. The trust, partnership, and familiarity created over the first few months were aided by the telerounds being at the bedside. |
| Feedback | Effects of Telemedicine ICU Intervention on Care Standardization and Patient Outcomes: An Observational Study | 2020 | Adult ICUs in two hospitals in United states | -Important elements are membership and administrative support, consensus on standardization of care, and periodic review of performance data. | Build NPS and platform feedback forms for the user and also our doctor, every end of round. | NPS assessments were implemented within the Mangará Digital software at the end of each round; in addition to checkpoint meetings with local coordinators every 2 months. |

| **Supplementary material 6. Description of selected studies in desk research (continuation)** | | | | | | |
| --- | --- | --- | --- | --- | --- | --- |
| **Topics** | **Title** | **Year** | **Context** | **Solutions/Insigts identified in the literature review** | **Solutions/ Insights identified in the Ideation process** | **Solutions Implemented** |
| Infrastructure | Key requirements of a video-call system in a critical care department as discovered during the rapid development of a solution to address COVID-19 visitor restrictions    PennMedicine (Available in  https://www.pennmedicine.org/for-patients-and-visitors/find-a-program-or-service/connected-health/virtual-visit)    EICU  Available in https://www.ummhealth.org/umass-memorial-health-all-hospitals/services-treatments/virtual-health-services/eicu    Philips ICU telemedicine program  (Available in https://www.usa.philips.com/healthcare/resources/landing/teleicu) | 2021  2022  2022  2022 | A tertiary referral hospital in  Galway, Ireland  Penn Medicine, Philadelphia, US  Hospitals in the UMass Memorial System, Massachusetts (US)  Philips eICU programs across the United States, Japan and Great Britain | - Comparison of the use of different video terminal technologies (Cart and tablet) for remote communication and market values. | Analysis of solutions in relation to pros and cons of usability and cost feasibility for the project. | The cart showed greater compatibility with the proposed journey, however, the tablet was the technology defined due to the lower cost and greater feasibility with regard to budget limitations |
| Collecting and Sharing Information | Identification of tele-ICU system requirements using a content validity assessment | 2016 | European intensive care professionals | The core functionality is to provide a data integration service that will be able to transform various data formats received from different subsystems (PDMS, electronic health records) to a central data format This is essential for data presentation in tele-ICU" | Pre-round information sharing every day in the morning. The front-end team would feed the platform with all exams and daily census. | Exam sharing is done during teleround orally or by showing the cell phone through the screen. But the daily census is placed pre or post round. We have not yet been able to build an exam submission feature within the platform. |

| **Supplementary material 6. Description of selected studies in desk research (continuation)** | | | | | | |
| --- | --- | --- | --- | --- | --- | --- |
| **Topics** | **Title** | **Year** | **Context** | **Solutions/Insigts identified in the literature review** | **Solutions/ Insights identified in the Ideation process** | **Solutions Implemented** |
| Collecting and Sharing Information | A Feasibility Study of Urgent Implementation of Cystic Fibrosis Multidisciplinary Telemedicine Clinic in the Face of COVID-19 Pandemic: Single-Center Experience | 2020 | University of Virginia (UVA), US | “Team members gather in a WebEx virtual workroom and fill out a clinic flow sheet/or patient follow-up sheet (maintained in a shared Google document [Google, Inc., Menlo Park, CA] with no identifiers of patient) with subspecialty members who will care for patients (respiratory therapist [RT], nutritionist [RD], social worker [SW], nursing coordinator and PsyD in addition to the physician)” | The use of documents/information shared by a cloud-based system with members of all specialties who will serve patients | A platform that allows the recording of care information in a holistic and multidisciplinary way and the sharing of information and goals with the professionals involved.. |
|  | Adaptation and validation of a multidisciplinary checklist for rounds in the intensive care unit | 2022 | Hospital in Paraná, Brazil | the daily use of a multidisciplinary checklist during the rounds (multidisciplinary bedside visit) contributes to increasing adherence to evidence-based practices.” | Building a structured instrument with important information for discussing cases in order to provide agility and objectivity in care. | The data collection flow was structured by systems and standardized for all patients, with a field for defining goals and sharing them with ICU professionals for follow-up |

| **Supplementary material 6. Description of selected studies in desk research (continuation)** | | | | | | |
| --- | --- | --- | --- | --- | --- | --- |
| **Topics** | **Title** | **Year** | **Context** | **Solutions/Insigts identified in the literature review** | **Solutions/ Insights identified in the Ideation process** | **Solutions Implemented** |
| People | Round multiprofissional em unidade de terapia intensiva: discussão para a implantação de procedimento operacional padrão    TeleICU Interdisciplinary Care Teams      Telemedicine in Intensive Care Units: Scoping Review | 2020  2019  2021 | Adult  ICU in a medium-sized hospital in the interior of  the state of Rio Grande do Sul, Brazil  This article examines the optimal TeleICU team composition, which is one that incorporates the use of an interdisciplinary approach, leverages technology, and is cognizant of varying geographic locations  A map of existing evidence on tele-ICU interventions, focusing on the analysis of the implementation context and identifying areas for further technological research | Studies do not suggest ideal team size, there is agreement that the care team has as its provider intensive care physicians (on duty), intensive care nurses and intensive care physiotherapists among others. | The team would be made up of a nurse, a physical therapist and a doctor, and we would have other professionals (phono, nutri, psycho) punctually in relerounds that needed it. | Today we have a nurse, a physiotherapist and a physician in each teleround. When another professional is needed, we invite a specialist from the hospital to participate in a specific teleround. |
