# Supplementary material for: User experience design methodologies for developing a tele-round platform in public intensive care units in northern and northeastern Brazil
Source: Front Digit Health. 2026 Apr 8;8:1713349. doi: 10.3389/fdgth.2026.1713349 (PMC13099869; doi:10.3389/fdgth.2026.1713349)
Supplement: Supplementary file 7 [file Supplementaryfile7.docx]

**Supplementary Material 7. Personas and Empathy Maps of “Physician” persona and persona and “Nurse” persona.
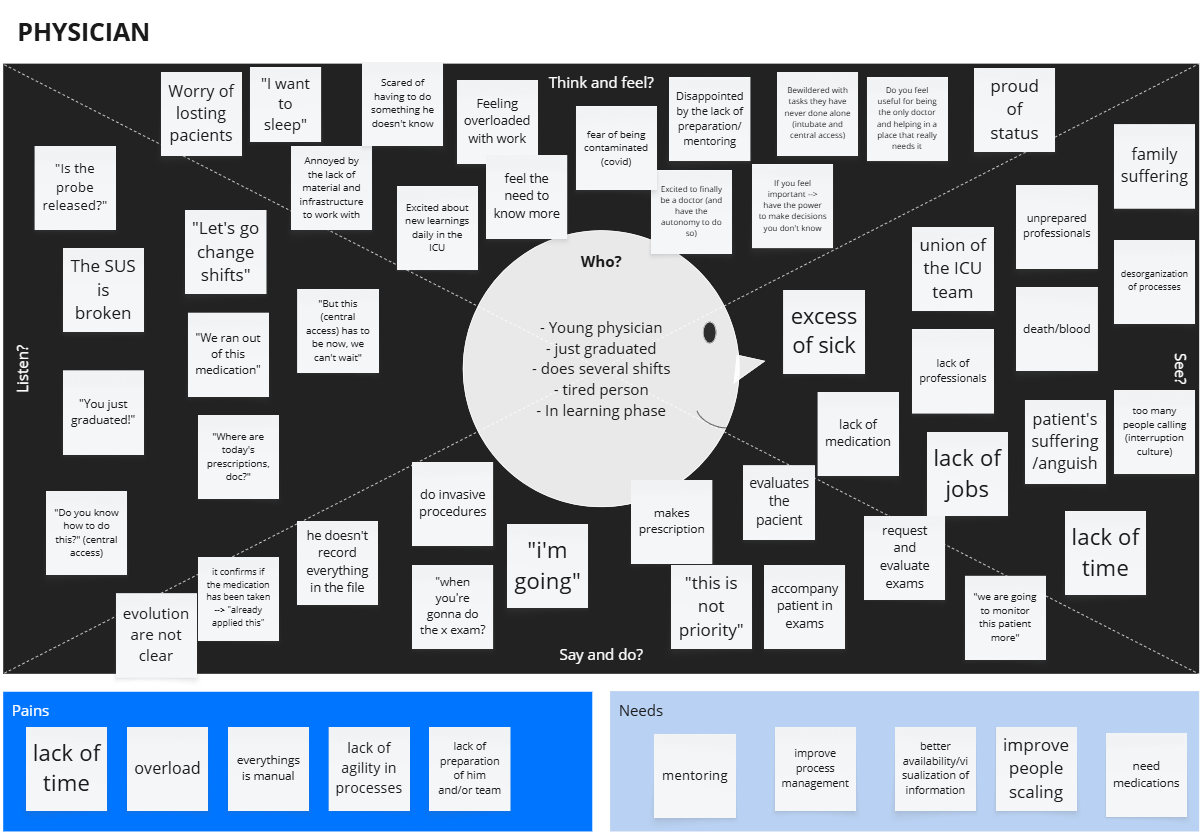
**

**
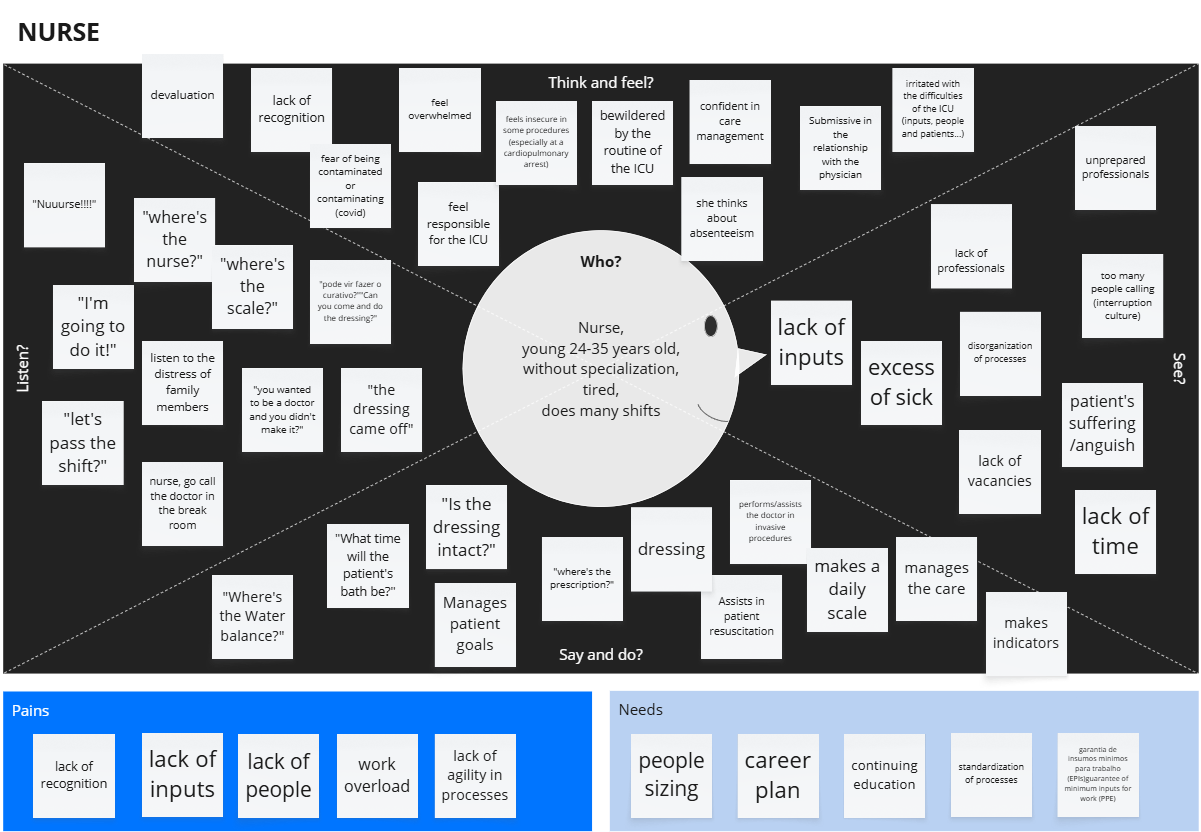
**
