# Supplementary material for: User experience design methodologies for developing a tele-round platform in public intensive care units in northern and northeastern Brazil
Source: Front Digit Health. 2026 Apr 8;8:1713349. doi: 10.3389/fdgth.2026.1713349 (PMC13099869; doi:10.3389/fdgth.2026.1713349)
Supplement: Supplementary file 8 [file Supplementaryfile8.docx]

| **Supplementary Material 8. Analysis of the infrastructure, processes, human resources and indicators of the ICUs that are part of the TeleUTI Project (N=10)** | | |
| --- | --- | --- |
| **Group** | **Characteristics** | **%** |
| **Physical infrastructure and Technology infrastructure** | **Number of hospital beds** |  |
|  | < 100 | 60 |
|  | > 100 | 40 |
|  | **Reference for COVID care** | 70 |
|  | **Surgical center** | 100 |
|  | **Exams performed in the hospital** |  |
|  | Laboratory | 100 |
|  | X-ray | 100 |
|  | Echocardiogram | 50 |
|  | ultrasound | 90 |
|  | Computed tomography | 70 |
|  | MRI | 0 |
|  | **Exams performed externally** |  |
|  | MRI | 80 |
|  | Computed tomography | 30 |
|  | Echocardiogram | 30 |
|  | **Interventional radiology (cardiac catheterization)** | 100 |
|  | **Interventional radiology (neurological)** | 90 |
|  | **Intermittent Hemodialysis** | 80 |
|  | **Continuous renal replacement therapy** | 50 |
|  | **Peritoneal dialysis** | 20 |
|  | **Protocols (Nosocomial infection and control commission)** | 100 |
|  | **Number of ICU beds** |  |
|  | ≤ 10 | 80 |
|  | > 10 | 20 |
|  | **Isolation beds available** | 100 |
|  | **Physical medical records** | 60 |
|  | **Hybrid medical records** | 40 |
|  | **Broadband internet available** | 80 |
|  | **Wi-Fi available in the ICU.** | 70 |
|  | **Internet outages** |  |
|  | Never | 20 |
|  | 1 time per month | 20 |
|  | 2-4 times per month | 50 |
|  | > 5 times per month | 10 |
|  | **Bedside RX** | 100 |
|  | **Bedside echocardiography** | 40 |
|  | **Bedside ultrasound** | 70 |
|  | **Number of ventilators** |  |
|  | < 10 | 10 |
|  | 10 a 15 | 60 |
|  | > 15 | 30 |

| **Supplementary Material 8. Analysis of the infrastructure, processes, human resources and indicators of the ICUs that are part of the TeleUTI Project (N=10) (continued)** | | |
| --- | --- | --- |
| **Group** | **Characteristics** | **%** |
| **Physical infrastructure and Technology infrastructure** | **Number of infusion pumps** |  |
|  | < 10 | 0 |
|  | 10 a 30 | 40 |
|  | > 30 | 60 |
|  | kit with defibrillator/cardioverter, medicines and emergency supplies (Exclusive for ICU) | 100 |
| **Human resources** | **Number of physicians** |  |
|  | < 10 | 60 |
|  | 10 a 15 | 30 |
|  | > 15 | 10 |
|  | **Number of nurses** |  |
|  | < 10 | 60 |
|  | 10 a 15 | 20 |
|  | > 15 | 10 |
|  | **Number of physiotherapists** |  |
|  | < 10 | 80 |
|  | 10 a 15 | 20 |
|  | > 15 | 0 |
|  | **Number of nursing technicians** |  |
|  | < 10 | 0 |
|  | 10 a 15 | 10 |
|  | > 15 | 90 |
|  | **Other professionals supporting the ICU team** |  |
|  | Speech therapist | 90 |
|  | Pharmacist | 80 |
|  | Nutritionist | 90 |
|  | Psychologist | 100 |
|  | Social worker | 90 |
|  | Multiparametric monitoring | 100 |
| **Processes** | **Standardization of continuous infusion drug solutions** | 80 |
|  | **Use instrument for shift handover** | 100 |
|  | **Multidisciplinary round/visit** | 90 |
|  | **Nursing Care Systematization (SAE)** | 100 |
|  | **Protocol for hand hygiene** | 90 |
|  | **Protocol on medication prescription, use and administration** | 90 |
|  | **Use bloodstream infection prevention bundle** | 90 |
|  | **Use urinary tract infection prevention bundle** | 90 |
|  | **Use ventilator-associated pneumonia prevention bundle** | 100 |
|  | **Protocol for pressure injury prevention** | 90 |
|  | **Daily census** | 100 |
| **Monitoring of indicators** | **Ventilator-associated pneumonia (VAP) incidence density** | 90 |
|  | **Incidence density of catheter-associated bloodstream infection** | 70 |
|  | **Incidence density of urinary tract infection associated with urinary catheter** | 90 |
|  | **Central Venous Catheter utilization rate** | 80 |
|  | **Urinary catheter utilization rate** | 80 |
|  | **Mechanical ventilation (MV) utilization rate** | 100 |
|  | **Pressure Ulcer Rate** | 80 |

| **Supplementary Material 8. Analysis of the infrastructure, processes, human resources and indicators of the ICUs that are part of the TeleUTI Project (N=10) (continued)** | | |
| --- | --- | --- |
| **Group** | **Characteristics** | **%** |
| **Monitoring of indicators** | **Tracheostomy rate** | 60 |
|  | **ICU mortality rate** | 100 |
|  | **Mortality rate (D28)** | 40 |
|  | **Length of stay in the ICU** | 90 |
|  | **Length of hospital stay** | 80 |

|  | | |
| --- | --- | --- |
|  |  |  |
|  |  |  |
|  |  |  |
|  |  |  |
|  |  |  |
|  |  |  |
|  |  |  |
|  |  |  |
|  |  |  |
|  |  |  |
|  |  |  |
|  |  |  |
|  |  |  |
|  |  |  |
|  |  |  |
|  |  |  |
|  |  |  |
|  |  |  |
|  |  |  |
|  |  |  |
|  |  |  |
|  |  |  |
|  |  |  |
|  |  |  |
|  |  |  |
|  |  |  |
|  |  |  |
|  |  |  |
|  |  |  |
|  |  |  |
|  |  |  |
|  |  |  |
|  |  |  |
|  |  |  |
|  |  |  |
|  |  |  |
|  |  |  |
|  |  |  |
|  |  |  |
|  |  |  |
|  |  |  |
|  |  |  |
|  |  |  |
