# Supplementary material for: User experience design methodologies for developing a tele-round platform in public intensive care units in northern and northeastern Brazil
Source: Front Digit Health. 2026 Apr 8;8:1713349. doi: 10.3389/fdgth.2026.1713349 (PMC13099869; doi:10.3389/fdgth.2026.1713349)

**Supplementary material 9. Wireframe of the main modules of teleround (Data does not include the details of any real patient)**

**
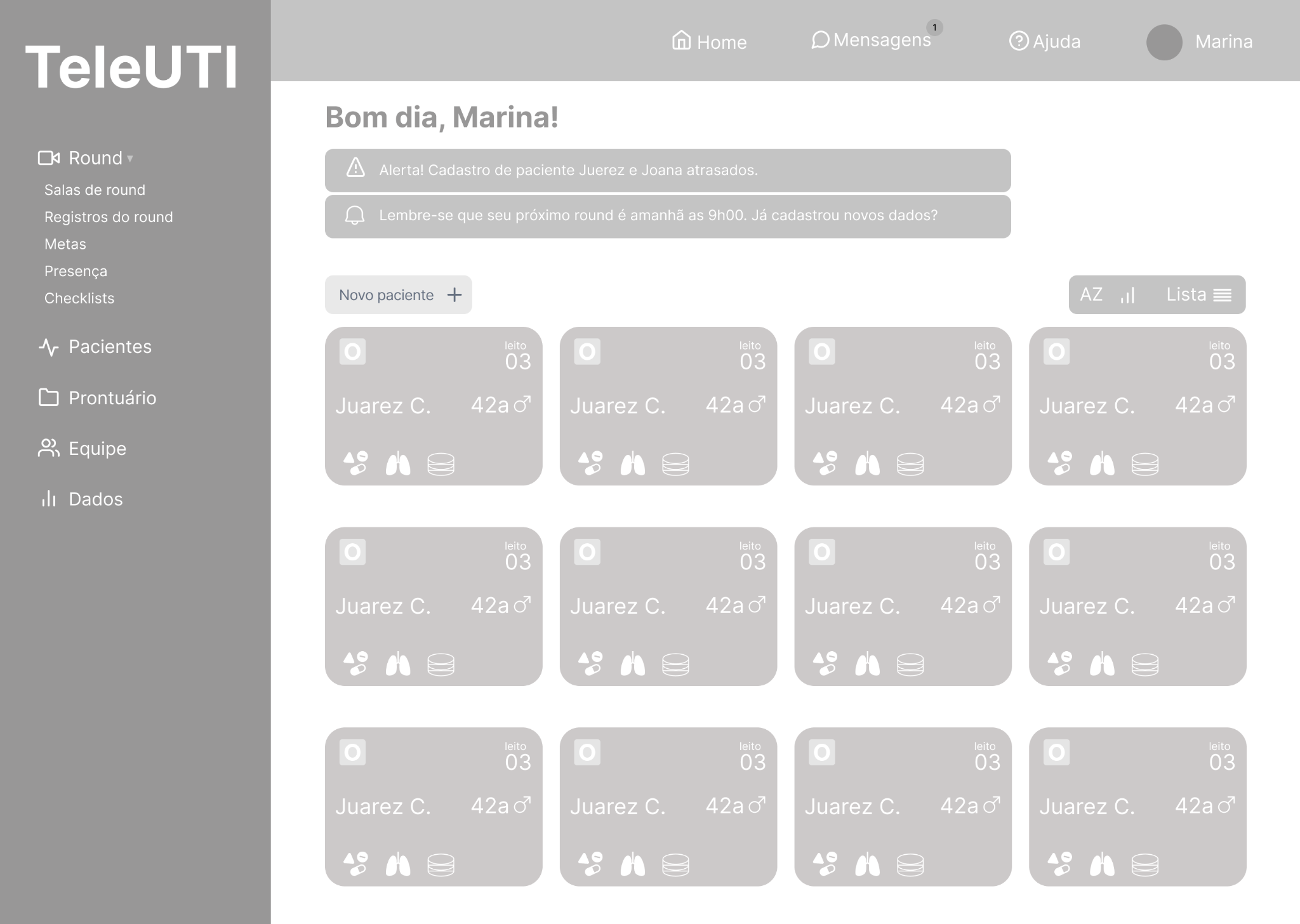
**


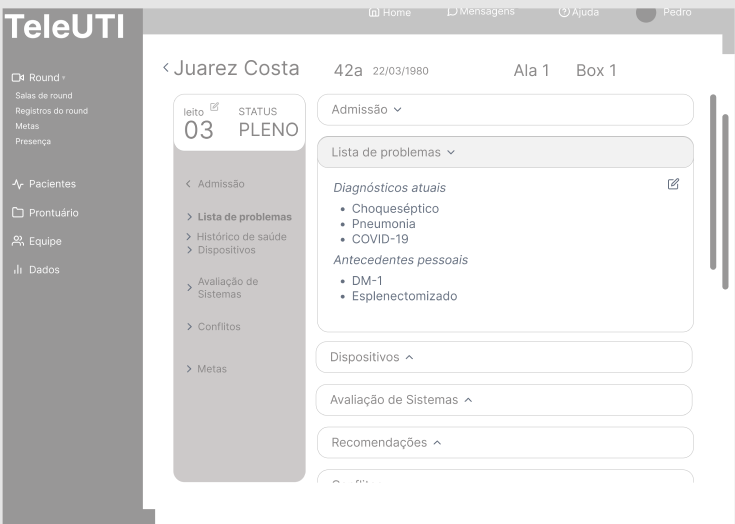


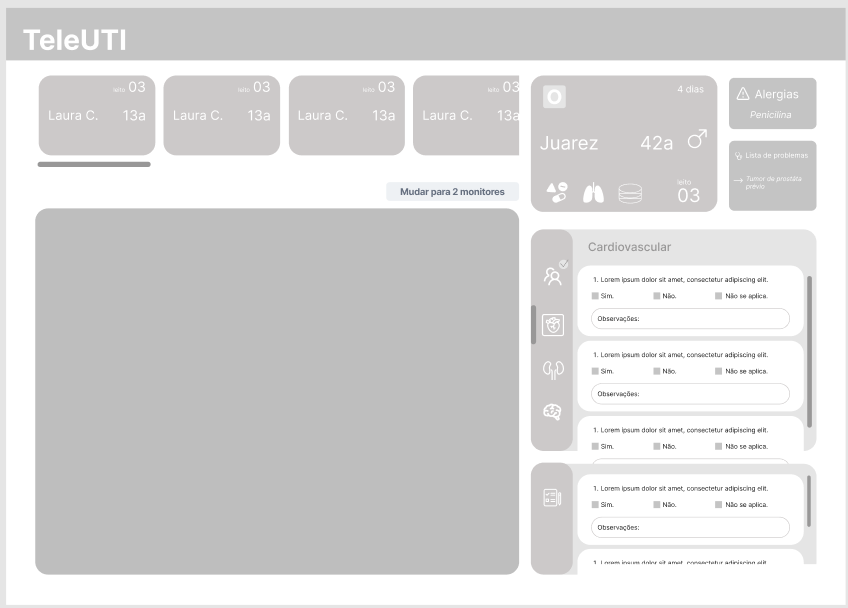


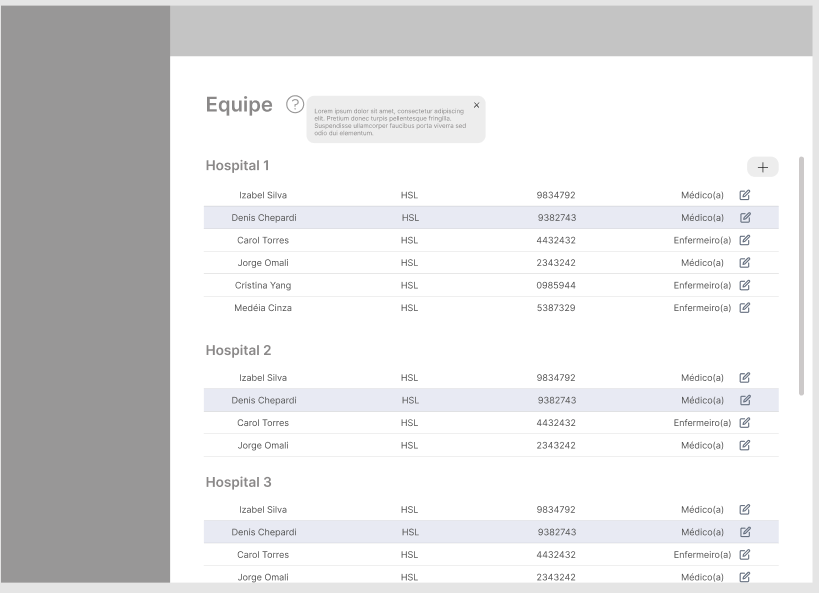


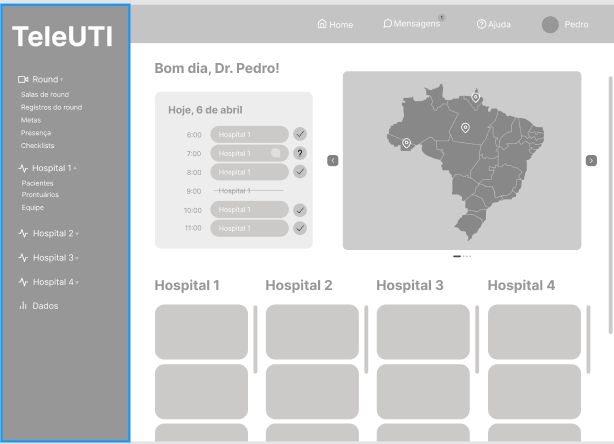


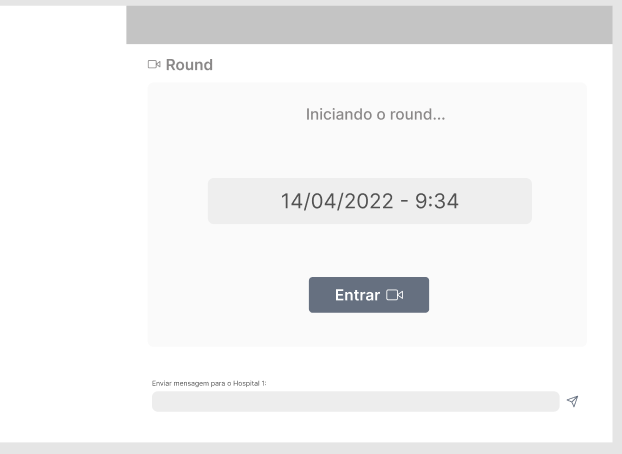

Supplement: Supplementary file 9 [file Supplementaryfile9.docx]
