# Supplementary material for: User experience design methodologies for developing a tele-round platform in public intensive care units in northern and northeastern Brazil
Source: Front Digit Health. 2026 Apr 8;8:1713349. doi: 10.3389/fdgth.2026.1713349 (PMC13099869; doi:10.3389/fdgth.2026.1713349)
Supplement: Supplementary file 10 [file Supplementaryfile10.docx]

**Supplementary material 10. Sitemap of the TeleUTI platform s.**

**
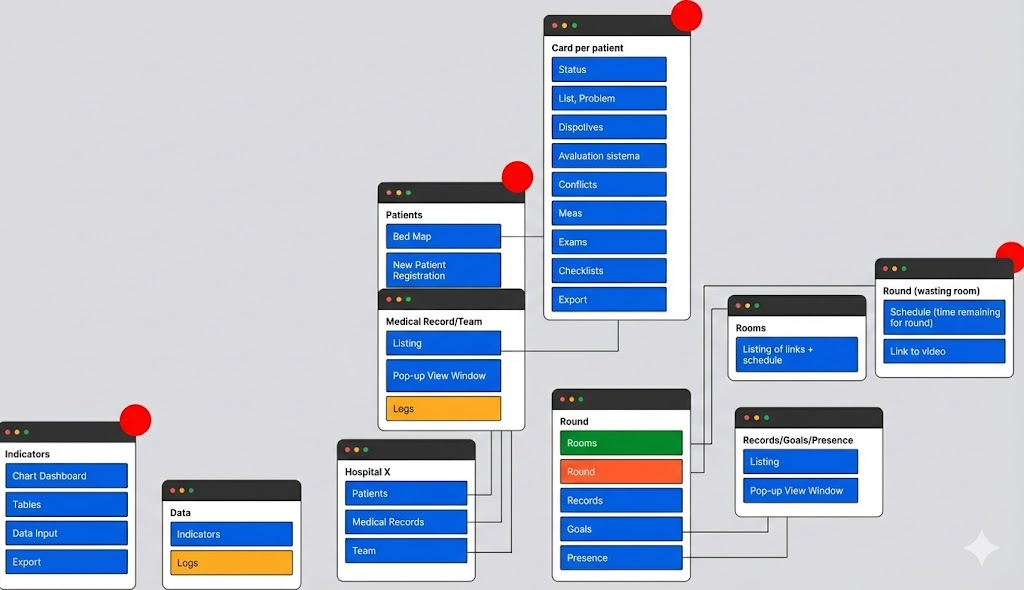
**

**
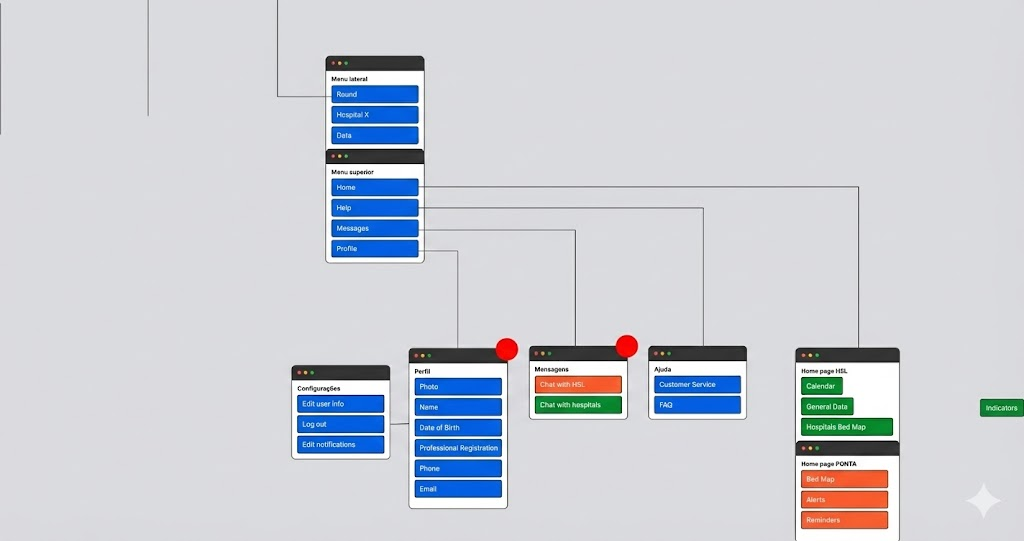
**

**Sitemap of the TeleUTI platform illustrating the information architecture derived from the Discover and Define phases of the Double Diamond framework. The diagram presents the hierarchical organization of core modules, including patient management, ICU bed mapping, tele-rounds, indicators, messaging, and user profile management. The sitemap reflects key design priorities identified during user research, such as rapid access to critical clinical information, role-based navigation, and reduction of cognitive load in time-constrained intensive care unit environments.**
