# Supplementary material for: User experience design methodologies for developing a tele-round platform in public intensive care units in northern and northeastern Brazil
Source: Front Digit Health. 2026 Apr 8;8:1713349. doi: 10.3389/fdgth.2026.1713349 (PMC13099869; doi:10.3389/fdgth.2026.1713349)
Supplement: Supplementary file 11 [file Supplementaryfile11.docx]

**Supplementary material 11. User flow representing a real-world clinical scenario in the TeleUTI platform. The diagram depicts the workflow of a bedside nurse registering a new ICU patient, accessing patient information, participating in a multidisciplinary tele-round, sharing diagnostic exams during the session, and accessing and printing the daily goals defined after the round. This flow operationalizes the “before, during, and after the tele-round” journey described in the Methods and Results sections and illustrates how the platform supports clinical decision-making, documentation, and continuity of care across different stages of use.**

**
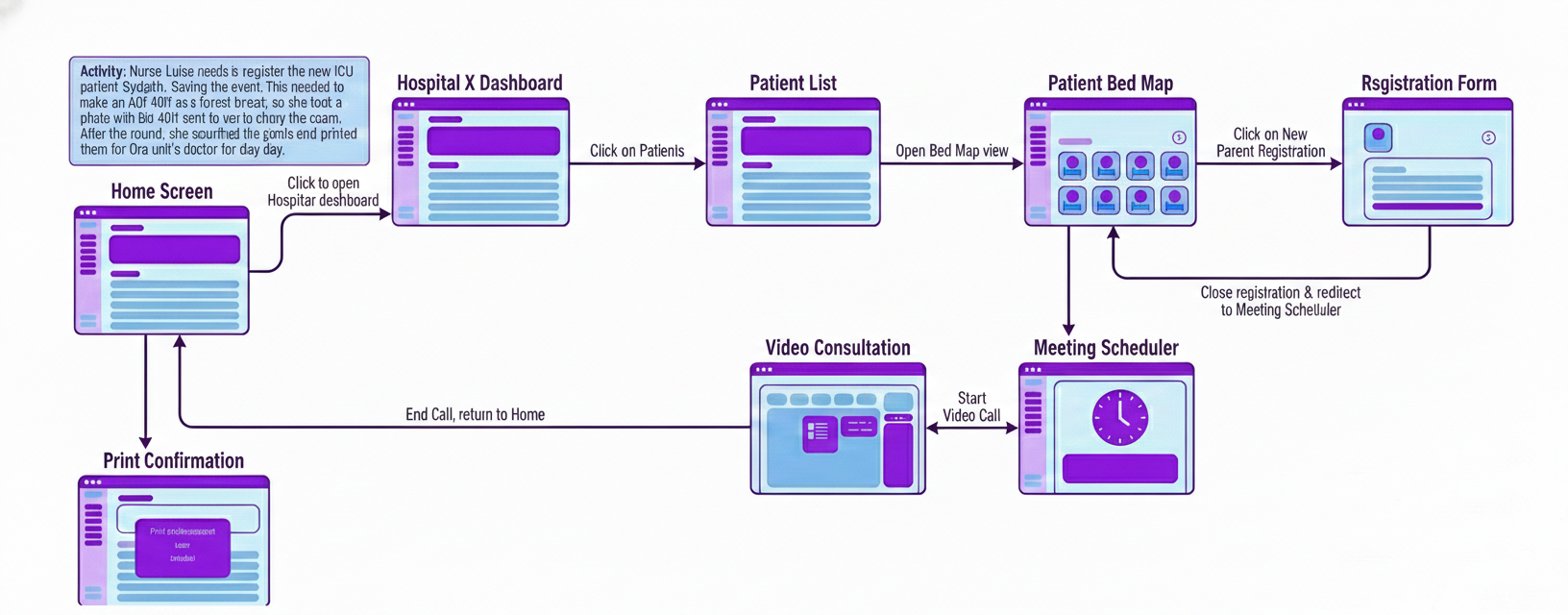
**
